# Three-year survival follow-up of patients with gastrointestinal cancer treated during the COVID-19 pandemic in Spain: data from the PANDORA-TTD20 study

**DOI:** 10.1093/oncolo/oyae300

**Published:** 2024-11-16

**Authors:** Pilar García-Alfonso, Paula Jimenez-Fonseca, Javier Soto-Alsar, Iosune Baraibar, Cristina Santos, Adelaida La Casta, Ismael Ghanem, Gema Pulido Cortijo, Axel Mariño Méndez, Roberto Pazo-Cid, Ruth Vera, Marcos Melián, Julia Alcaide, Begoña Graña, David Páez, Inmaculada Gallego, Miriam Lobo, Miguel Borregón, Ana Fernández Montes, Eva Martínez de Castro, Alberto Carmona-Bayonas, Enrique Aranda

**Affiliations:** Medical Oncology Department, Hospital General Universitario Gregorio Marañón, Instituto de Investigación Sanitaria Gregorio Marañón (IiSGM), Universidad Complutense, Madrid, 28007, Spain; Medical Oncology Department, Hospital Universitario Central de Asturias (HUCA), Instituto de Investigación Sanitaria del Principado de Asturias (ISPA), Oviedo, 33011, Spain; Medical Oncology Department, Hospital General Universitario Gregorio Marañón, Instituto de Investigación Sanitaria Gregorio Marañón (IiSGM), Universidad Complutense, Madrid, 28007, Spain; Medical Oncology Department, Vall d’Hebron Barcelona Hospital Campus, Vall d’Hebron Institute of Oncology (VHIO), Universitat Autónoma de Barcelona, CIBERONC, Barcelona, 08035, Spain; Medical Oncology Department, Institut Català d’Oncologia (ICO), Translational Research Laboratory, ICO-Bellvitge Biomedical Research Institute (IDIBELL)-CIBERONC, Barcelona, 08908, Spain; Medical Oncology Department, Hospital Universitario de Donostia, Guipúzcoa, 20014, Spain; Medical Oncology Department, Hospital Universitario La Paz, Madrid, 28046, Spain; Medical Oncology Department, Hospital Universitario Reina Sofía, Universidad de Córdoba, Instituto Maimónides de Investigación Biomédica de Córdoba (IMIBIC), CIBERONC, Instituto de Salud Carlos III (ISCIII), Córdoba, 14004, Spain; Medical Oncology Department, HUCA, ISPA, Oviedo, 33011, Spain; Medical Oncology Department, Hospital Universitario Miguel Servet, Aragon Institute of Biomedical Research (IISA), Spanish Cancer Network (RTICC), ISCIII, Zaragoza, 50009, Spain; Medical Oncology Department, Hospital Universitario de Navarra, Pamplona, 31008, Spain; Medical Oncology Department, Instituto Valenciano de Oncología (IVO), Valencia, 46009, Spain; Medical Oncology Intercenter Unit, Regional and Virgen de la Victoria University Hospitals, Instituto de Investigación Biomédica de Málaga (IBIMA), Málaga, 29010, Spain; Medical Oncology Department, Complejo Hospitalario Universitario de A Coruña (CHUA), Instituto de Investigación Biomédica de A Coruña (INIBIC), Coruña, 15006, Spain; Medical Oncology Department, Hospital Santa Creu i Sant Pau, Barcelona, 08041, Spain; Medical Oncology Department, Hospital Universitario Virgen del Rocío, Instituto de Biomedicina de Sevilla (IBIS), Sevilla, 41013, Spain; Medical Oncology Department, Hospital General Universitario de Valencia, Valencia, 46014, Spain; Medical Oncology Department, Hospital General Universitario de Elche, Elche, 03203, Spain; Medical Oncology Department, Complejo Hospitalario Universitario de Orense (CHUO), Orense, 32005, Spain; Medical Oncology Department, Hospital Universitario Marqués de Valdecilla, Instituto De Investigación Marqués de Valdecilla (IDIVAL), Santander, 39008, Spain; Medical Oncology Department, Hospital Universitario Morales Meseguer, Universidad de Murcia, Instituto Murciano de Investigación Biosanitaria (IMIB), Murcia, 30008, Spain; Medical Oncology Department, Hospital Universitario Reina Sofía, Universidad de Córdoba, Instituto Maimónides de Investigación Biomédica de Córdoba (IMIBIC), CIBERONC, Instituto de Salud Carlos III (ISCIII), Córdoba, 14004, Spain

**Keywords:** gastrointestinal cancer, coronavirus disease 2019, Spain, PANDORA-TTD20, RETUD registry, spatial frailties

## Abstract

**Introduction:**

The initial SARS-CoV-2 pandemic wave in Spain in 2020 precipitated significant paradigm shifts in gastrointestinal oncology patient management. This study captures the “Zeitgeist” of this period by analyzing adaptive strategies, treatment modifications, and survival outcomes, leveraging a 3-year follow-up perspective to extract insights from this unprecedented experience.

**Methods:**

We conducted a multicenter, retrospective cohort study utilizing the RETUD-TTD registry, encompassing 703 patients across 19 Spanish centers in April 2020. We evaluated alterations in clinical practice, therapeutic approaches, coronavirus disease 2019 (COVID-19)-related impacts, and patient survival. A Bayesian hierarchical model was employed to identify potential regional-specific frailties.

**Results:**

The peak of the pandemic in April 2020 catalyzed substantial shifts in oncological care delivery. Outpatient consultations decreased by 13%, with a notable selection bias toward cases with more favorable prognostic indicators. Multidisciplinary tumor board discussions were significantly curtailed (eg, mean monthly colorectal cancer cases discussed was reduced from 40 to 23), compromising qualitative care measures. This occurred concurrently with an average of over 3 oncologists per center on medical leave. Contrary to initial concerns, the healthcare system demonstrated remarkable resilience. The majority of patients received standard-of-care therapies with regulatory approval, albeit with regimen modifications in 15% of cases. These adaptations included extended dosing intervals, dose intensity modulations, and transitions to oral formulations while maintaining unexpectedly stable long-term survival outcomes. The Bayesian frailty model detected minimal unmeasured prognostic factors related to geographic location, and the type of pandemic-induced adaptation did not significantly impact survival. The model revealed that coronavirus disease 2019’s impact was less pronounced than other core prognostic variables.

**Conclusions:**

The decentralized Spanish healthcare system exhibited substantial robustness in managing pre-pandemic diagnosed gastrointestinal malignancies, despite asymmetrical, and occasionally severe organizational disruptions. The insights gleaned from this experience could inform future crisis preparedness strategies and optimize care provision during subsequent public health emergencies.

**Implications for practice**

The impact of coronavirus disease 2019 (COVID-19) on gastrointestinal cancer care in Spain was examined, revealing a “test laboratory” scenario. This study highlighted asymmetric changes in treatment strategies during the pandemic. Despite challenges, most centers maintained oncology care, witnessing only a slight 13% consultation drop. The data showed stable survival outcomes, mirroring figures from the pre-COVID-19 era. Spain’s healthcare displayed strong resilience, emphasizing the adaptability of its systems in crisis.

## Introduction

Spain was severely affected by the coronavirus disease 2019 (COVID-19) pandemic in 2020, ranking third in Europe for confirmed cases with nearly 30 000 deaths.^1-3^ An estimated 6% of the population was infected during the first 2 waves, resulting in over 100 000 hospital admissions.^3,4^ The impact varied regionally,^5,6^ with areas like Madrid and Catalonia experiencing higher case fatality rates.^6-9^

The nationwide state of emergency, declared in March 2020, led to regional lockdowns^10^ and a decentralized crisis management approach.^3^ This stressed the healthcare system, hampering its capacity to diagnose, and treat non-COVID-19 diseases.^11^ Cases of chronic pathologies, including neoplastic diseases, decreased markedly during the first 2 waves.^12-14^

Gastrointestinal cancer screening in Spain was significantly disrupted, leading to many undiagnosed patients. Compared to 2019, diagnoses of digestive cancers,^15-17^ including colorectal cancer, fell by 17%.^18^ This underdiagnosis persisted in some regions until 2022.^19^ High COVID-19 mortality among oncology patients led to hesitancy in seeking medical consultation,^20^ exacerbated by longer waiting times for diagnostic tests and reduced surgical activity.^17,21-23^

The impact on Medical Oncology Services, particularly on perioperative cancer management and treatment in advanced age, has not been thoroughly documented. New visits to medical oncology departments dropped by around 20% between February and June 2020 across Spain.^17^ Management protocols for advanced tumors were modified swiftly, promoting preoperative therapies to delay surgeries,^22^ and telemedicine consultations became more prevalent.^24^ Scientific societies published consensus documents on cancer patient management, particularly for gastrointestinal tumors.^25-27^ However, there was a noticeable decline in medical day hospital activity, including lower administration of chemotherapy and a 13% reduction in clinical trial recruitment.^17^

This study aims to document the alterations in structure and care provision for gastrointestinal cancer in Spain’s oncology departments affiliated with a national cooperative group. It evaluates the impact of pandemic-related policies on research, treatment, and survival across different geographical regions of Spain during the peak of the pandemic in April 2020. The findings will provide insights into healthcare resilience and adaptations in oncology care during unprecedented disruptions.

## Method

This study utilized data from the Spanish Registry of Digestive Tumors (RETUD), establishing PANDORA-TTD20 as a retrospective cohort involving 19 TTD-affiliated centers. The study population comprised patients seen between April 20, 2000 and 24, 2020, during the peak of Spain’s first COVID-19 wave. Eligible participants were adults diagnosed with gastrointestinal cancer who were treated at the Oncology Department. Outcome analyses focused on patients with localized tumors under active treatment and all patients with advanced disease stages. A 3-year survival assessment was conducted with a follow-up in April 2023. An additional survey investigated staffing, tumor committee activity, and patient volume in participating centers. The primary aim was to examine changes in organization, structures, staffing, and committees that affect digestive cancer management, and identify potential spatial frailties associated with regional disparities.^28^ The primary endpoint was overall survival (OS) from April 22, 2020, to any-cause death or censoring. Secondary aims sought to gain insight into cancer management patterns during the pandemic. Variables included medical factors (ECOG-PS, comorbidities, age, sex, disease stage, tumor location, treatment goals, clinical trial participation), management patterns, SARS-CoV-2 infection rates and consequences, center-specific data, and regional COVID-19 information from the Spanish Ministry of Health.^3^ Statistical analysis employed a Bayesian semiparametric proportional hazards model, with spatial location modeled via frailties using an independent identically distributed non-informative Gaussian prior.^28,29^ An additional analysis used a Gaussian random field prior based on geographic coordinates.^30^ A multivariable model incorporated all mentioned covariates. Exponentiated frailties were represented on a geographic map, with correlation between regional frailties and COVID-19 situation quantified using Tjostheim’s coefficient.^31^ Basic summary statistics, Kaplan-Meier estimator for survival modeling, and 2-tailed Wilcoxon test for paired data were also utilized. Analyses were performed using R v4.3.1 with relevant libraries.^30,32-34^ The study adhered to ethical regulations, obtained informed consent from living participants and received approval from the Drug Research Ethics Committee of all participating centers. A comprehensive description of the materials and methods employed in this study, including detailed protocols, statistical analyses, and Supplementary Data, is provided in the Supplementary Appendix.

## Results

### Patient care during the pandemic

A cohort of 703 patients, seen in-person at 19 participating Medical Oncology services between April 20, 2000 and 24, 2020, was consecutively recruited for the PANDORA-TTD20 study. Table 1 provides baseline characteristics, with Supplementary Table S1 showing study recruitment by participating regions and centers and Supplementary Tables S3 and S4 depicting participating oncology departments’ characteristics and patient baseline characteristics per center, respectively. Of the cohort, 31% (*n* = 221) had localized tumors, with 55% (121/221) of those under follow-up without active intervention. Of the patients with localized cancers requiring evaluation (100/221), 79% (79/100) were recommended for systemic antineoplastic treatment. Of those recommended, 93% (74/79) received the treatment. Standard adjuvant therapy was administered to 95% (70/74) of treated patients, with 18% (13/74) undergoing regimen modifications. Oncology consultation appointments were affected in 9% of cases (Table 2). Of the rectal cancer surgeries (*n* = 16), one case (6%) was postponed by 3 weeks.

**Table 1.** Baseline characteristics (April 2020)

Baseline characteristics	*N* (%)
Age, mean (range)	65 (30-89)
Sex, women	260 (36.9)
Performance status, ECOG-PS	
0	209 (29.7)
1	341 (48.1)
2	66 (9.3)
3	16 (2.2)
4	5 (0.7)
Unknown	66 (9.3)
Comorbidities that limit systemic treatment *	128 (18.1)
Patient referred from another area or health center	138 (19.6)
Primary cancer site	
Esophagus	41 (5.8)
Stomach	47 (6.6)
Pancreas	150 (21.3)
Liver and bile duct	64 (9.1)
Colon	266 (37.8)
Rectum	127 (18.0)
Anus	8 (1.1)
Tumor stage	
Non metastatic	221 (31.4)
Metastatic	482 (68.5)
Clinical trial participants	76 (10.8)

**Table 2.** Management of patients with non-metastatic or metastatic cancer during the COVID-19 pandemic (April 2020).

Management of patients with non-metastatic cancer	*N* (%) 221 (100)
Treatment and follow-up management	
** **Follow-up visits	121 (54.7)
** **Treatment visits	100 (45.3)
** **Adjuvant treatment not indicated	21 (9.5)
** **Adjuvant chemotherapy indicated by oncologic criteria	79 (35.7)
** **Prescribed despite the pandemic	74 (33.4)
** **Not prescribed due to the pandemic	1 (0.45)
** **Not prescribed for other reasons	4 (1.8)
Treatment strategies	
** **Standard regimen	70 (31.76)
** **Modification of the standard regimen	13 (5.9)
** **Standard regimen adjusting dose	6 (2.7)
** **Standard regimen adjusting interval	5 (2.2)
** **Standard regimen adjusting dose and interval	1 (0.4)
** **Change the route of administration of any drug from IV to oral	1 (0.4)
** **Modification of visits	21 (9.5)
** **In-person and telephone visits were alternated	13 (5.8)
** **Only telephone visits were conducted	6 (2.7)
** **Visits were spaced out	2 (0.9)
Management of patients with metastatic cancer	*N* (%) 482 (100)
Treatment and follow-up management	
** **Follow-up visit	109 (22.6)
** **Decision on treatment	373 (87.4)
** **Alternative to systemic treatment *	60 (12.5)
** **Systemic treatment indicated by medical criteria	313 (64.9)
** **Prescribed despite the pandemic	272 (56.4)
** **Not prescribed due to the pandemic	7 (1.4)
** **Not prescribed for other reasons	33 (6.8)
Reasons for not initiating or continuing systemic treatment	
** **Improved supportive care as systemic treatment is contraindicated	10 (2.0)
** **Improved supportive care favored by the pandemic	8 (1.6)
** **Mixed reasons	23 (4.7)
Type of systemic treatment	*N* (%) 272 (100)
First-line chemotherapy with palliative intention	126 (46.3)
First-line chemotherapy with intention to convert	40 (14.7)
Second-line chemotherapy	67 (24.6)
Third-line chemotherapy or beyond	31 (11.4)
Adjuvant chemotherapy for metastasis resection	8 (2.9)
Treatment strategies	
** **Standard therapy	222 (81.7)
** **Modification of the regimen	50 (18.3)
** **Alternative antineoplastic agents to the standard	9 (1.8)
** **Standard regimen adjusting dose	15 (3.1)
** **Standard regimen adjusting interval	17 (3.5)
** **Standard regimen adjusting dose and interval	6 (1.2)
** **Change the route of administration of any drug from IV to oral	3 (0.6)
** **Modification of visits	51 (18.7)
** **Several cycles were scheduled without visits and without blood tests	3 (0.6)
** **In-person and telephone visits were alternated	31 (6.4)
** **Only telephone visits were conducted	3 (0.6)
** **Visits were spaced out	16 (3.3)
Suitability for metastasis surgery	27 (9.9)
** **Performed as scheduled	20 (4.1)
** **Delayed due to COVID-19	4 (0.8)
** **Chemotherapy was continued	4 (0.8)
** **Rejected due to disease progression	1 (0.2)
** **Replaced by locoregional treatments (yttrium microspheres)	2 (0.4)

Metastatic disease was present in 68% (482/703), with 65% (313/482) of those indicated for active treatment. Despite challenges, 87% (272/313) initiated or continued treatment. The pandemic was the sole reason for stopping therapy in 2% (7/313) of cases. Among treated patients (*n* = 272), pandemic-related adjustments included: lengthened intervals among cycles (6%, 17/272), dose adjustments (5%, 15/272), both modifications (2%, 6/272), and switching to oral forms (1%, 3/272). Appointments were transitioned to telephone consultations in 11% (31/272) and spaced out for 6% (16/272) of cases. Table 2 lists these modifications.

Regarding clinical research, 18% (56/313) of eligible patients could not participate in trials due to pandemic-linked causes. Of pre-enrolled patients (*n* = 76), 84% (64/76) maintained experimental treatment, 14% (11) withdrew due to progression, and one discontinued due to the pandemic. For metastatic cancer patients eligible for surgery (9%, 27/313), 74% (20/27) underwent the operation as planned. Supplementary Table S5 provides a detailed overview of metastatic cancer patient management per center.

By April 20–24, 2020, 4.3% (30/703) of subjects had acquired COVID-19, with 83% (25/30) having metastatic cancer. The infection-related mortality was 7% (2/30). COVID-19 indirectly impacted patient care, with 30% (9/30) experiencing progression after treatment interruption, and 23% (7/30) requiring treatment adjustments or halting. Supplementary Table S6 details COVID-19’s impact on these patients.

### Organizational changes and status of centers

The characteristics of the 19 centers are displayed in Supplementary Table S3. Of these, 17 serve population areas exceeding 300 000 inhabitants. Typically, medical oncology departments have a median of 28 oncologists (range 16-65), with 5 (range 2-12) specializing in digestive tumors. Twelve hospitals had specific committees for pancreatohepatobiliary, colorectal, and esophagogastric tumors, while 7 had a single gastrointestinal tumor committee.

In April 2020, centers averaged 3.2 COVID-19-related absences (range 0-9), with 2.3 due to staff contracting COVID-19 and 0.8 (range 0-4) due to preventive quarantine. Total leaves increased from 0.4 to 3.8 per center between February and April 2020 (Wilcoxon test, *P* = .0016). Adaptations are shown in Supplementary Table S7. Among centers, 37% (*n* = 7) maintained regular activity, 58% (*n* = 11) made adjustments, and 5% (*n* = 1) suspended many oncological activities. Supplementary Table S8 presents patient characteristics grouped by adaptation type.

**Figure 1.**
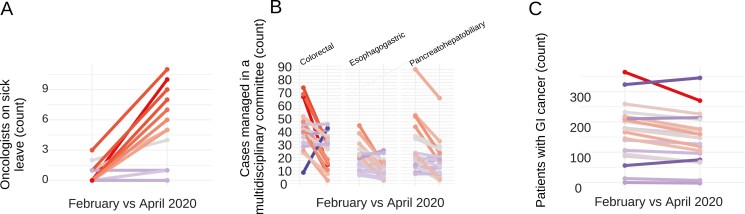
Oncologist leaves and patient management in committees and consultations during the COVID-19 pandemic. The colors and gradient of the arrows indicate the intensity of the changes.

**Figure 2.**
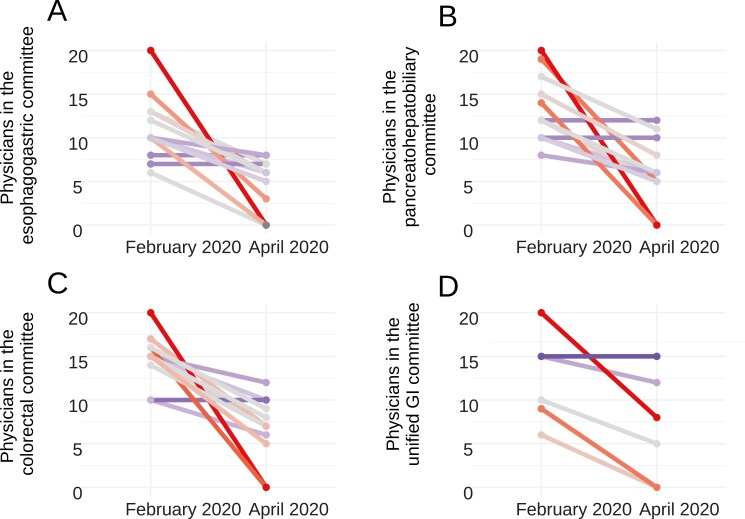
Staff involvement in multidisciplinary committees for patients with gastrointestinal cancer: a comparison of February and April 2020. The colors and gradient of the arrows indicate the intensity of the changes.

Staff relocation occurred at 58% (11/19) of the centers (Figure 1). Patient consultations decreased by 13% from February to April 2020 (176-153, Wilcoxon test, *P* = .002). Multidisciplinary committee meetings saw significant declines (Wilcoxon test, *P* < .01): colorectal committees from 40 to 23 cases, esophagogastric from 17 to 9, and pancreatohepatobiliary from 24 to 16 (Figure 1B). Committee participation also dropped, with colorectal tumor committees decreasing from 14.8 to 6.7 specialists per center (Wilcoxon test, *P* = .008). Only 3/12 colorectal cancer committees continued in-person meetings, and similar trends were observed in other committees (Figure 2).

In gastroesophageal tumor committees, 4/12 switched to non-face-to-face meetings, and 3/12 alternated. Meeting frequency changed from weekly to every 2-4 weeks, conducted primarily via video conference. Regarding management protocols, 58% (11/19) of centers adopted SEOM recommendations for COVID-19, 16% (3/19) followed ESMO guidelines, and 26% (5/19) developed their own protocols.

### Survival-based outcomes and frailties

**Figure 3.**
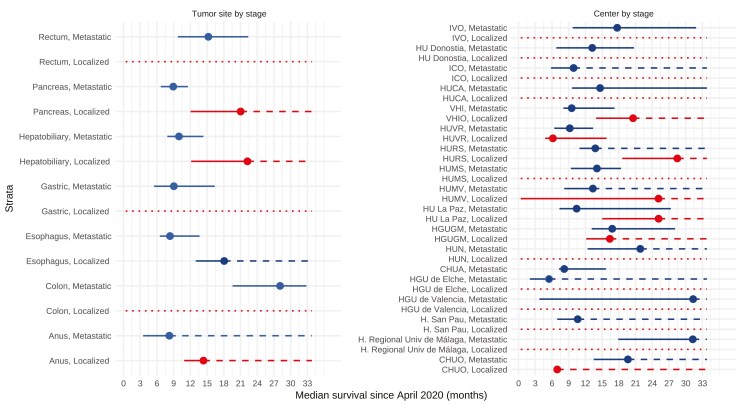
Overall survival stratified by tumor type, center, and stage (unadjusted). Abbreviations: CHUA, Complejo Hospitalario Universitario de A Coruña; CHUO, Complejo Hospitalario Universitario de Orense; H. San Pau, Hospital de la Santa Creu i Sant Pau; HGU de Elche, Hospital General Universitario de Elche; HGU de Valencia, Hospital General Universitario de Valencia; HGU, Hospital General Universitario; HGUGM, Hospital General Universitario Gregorio Marañón; HU La Paz, Hospital Universitario La Paz; HU, Hospital Universitario; HUCA, Hospital Universitario Central de Asturias, HUMS, Hospital Universitario Miguel Servet; HUMV, Hospital Universitario Marqués de Valdecilla; HUN, Hospital Universitario de Navarra; HURC, Hospital Universitario Ramón y Cajal; HURS, Hospital Universitario Reina Sofía; HUVR, Hospital Universitario Virgen del Rocío ICO L’Hospitalet, Instituto Catalán de Oncología; IVO, Instituto Valenciano de Oncología; VHIO, Hospital Universitario de la Vall d’Hebron y Vall d’Hebron Instituto de Oncología;. Dashed lines represent the upper limits of the confidence intervals that were not reached. The dotted lines indicate that there are no available events for that stratum. The color red identifies patients with localized cancer, and the color blue identifies patients with metastatic cancer. Hospital Universitario Ramón y Cajal provided only organizational information but did not report direct patient data.

Median survival rates by tumor type, center, and stage over 3 years are shown in Figure 3 and Supplementary Table S9. No definitive association was found among organizational adaptations, committee modifications, and survival outcomes.

**Figure 4.**
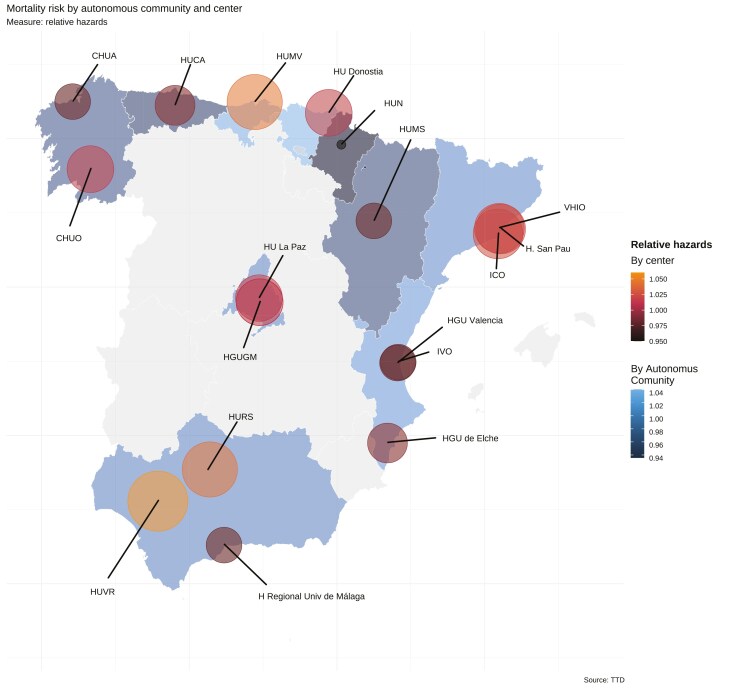
Mortality risk by autonomous community and center. Abbreviations: CHUA, Complejo Hospitalario Universitario de A Coruña; CHUO, Complejo Hospitalario Universitario de Orense; H. San Pau, Hospital de la Santa Creu i Sant Pau; HGU de Elche, Hospital General Universitario de Elche; HGU de Valencia, Hospital General Universitario de Valencia; HGU, Hospital General Universitario; HGUGM, Hospital General Universitario Gregorio Marañón; HU La Paz, Hospital Universitario La Paz; HU, Hospital Universitario; HUCA, Hospital Universitario Central de Asturias, HUMV, Hospital Universitario Marqués de Valdecilla; HUMS, Hospital Universitario Miguel Servet; HUN, Hospital Universitario de Navarra; HURC, Hospital Universitario Ramón y Cajal; HURS, Hospital Universitario Reina Sofía; HUVR, Hospital Universitario Virgen del Rocío.; ICO L’Hospitalet, Instituto Catalán de Oncología; IVO, Instituto Valenciano de Oncología; VHIO, Hospital Universitario de la Vall d’Hebron y Vall d’Hebron Instituto de Oncología*Note*: The bluer or more orange it is, the higher the hazard rate. Hospital Universitario Ramón y Cajal provided only organizational information but did not report direct patient data.

A spatial multivariable model revealed minimal variations in hazard rates associated with geographical locations, both regionally and center-wise (Figure 4), with frailties approaching zero. This suggests a low probability of unobserved variables influencing prognosis. The effect of known covariables is presented in Supplementary Table S10, showing the expected association of ECOG-PS, line number, and stage with prognosis. Supplementary Figure S1 illustrates the posterior probability distribution for hazard ratios in each autonomous community, emphasizing the relative uniformity among regions.

Data showed minimal correlation with regional COVID-19 incidence, with a Tjostheim’s coefficient of −0.056 (standard error, 0.33). Supplementary Figure S2 displays the regional distribution of cumulative COVID-19 incidences in April 2020. In a multivariable Cox model, adaptation type did not significantly influence survival (*P* = .346), while the main prognostic factors were ECOG-PS, tumor location, line of treatment, and stage.

## Discussion

This study investigated the varying impact of COVID-19 on gastrointestinal cancer management across Spain’s regions. The pandemic significantly transformed organizational structures, affecting staffing, consultation modalities, and committee discussions.^35^ Despite these disruptions, most centers maintained oncology patient care with necessary adjustments.

During April 20–24, 2020, patient consultations decreased by 13% compared to February 10–14, 2020. This reduction was achieved by prioritizing patients with better functional status, evidenced by the absence of ECOG-PS > 2 cases in 8 centers, cases with complicating comorbidities in 4 centers, and patients eligible for clinical trials in 6 hospitals. Most patients eligible for treatment received care without substantial alterations, even during the pandemic’s peak.

OS outcomes remained like pre-pandemic historical controls. Median OS from April 2020 was 28.1 months (95% CI, 19.6-32.8) for advanced colorectal cancer, comparable to 27.6 months (95% CI, 25.9-29.2) in the 2018-2019 PROMETEO registry.^36,37^ For advanced gastric cancer, median OS was 9 months (95% CI, 5.5-16.4), similar to the pre-pandemic AGAMENON-SEOM registry’s 10.8 months (95% CI, 10.5-11.1) (*Z*-test, *P* = .518).^36^ For advanced pancreatic cancer, median OS was 8.9 months (95% CI, 6.7-11.5), aligning with the ANICE-PAC study’s 7.2 months (95% CI, 6.0-8.5).^38^ For localized esophageal cancer, our findings of 18.1 months (95% CI 13—not reached) were not significantly different from the AGAMENON registry’s 22.5 months (95% CI, 18.5-31.1).^39^ The most significant change was the reduction in multidisciplinary committee evaluations; however, the impact on prognosis appears minimal, as decisions were made through alternative channels that ensured process efficacy.

Our results reveal stable OS outcomes in cancer patients treated during the first COVID-19 wave peak, highlighting the Spanish healthcare system’s resilience. This contrasts with previously described trends in diagnostic management.^40,41^ Literature has suggested that the pandemic underscored chronic issues in the system’s decentralized structure,^15-17^ with decreased attention during initial cancer diagnostic phases documented across healthcare domains.^42,43^ Patient surveys indicated a perceived decline in care,^44^ yet this trend is not confirmed in our study of pre-pandemic diagnosed patients with gastrointestinal cancer. After a 3-year follow-up, we found no definitive evidence linking survival outcomes with organizational adaptations, committee modifications, or therapeutic decisions at each center. Analysis of spatial frailties^45^ suggests that regional disparities due to unobserved variables are unlikely to have influenced patient prognosis. Adjustments and impacts were relatively uniform across regions, with mechanisms mitigating effects on most patients.

Limitations include the retrospective design, albeit strengthened by a 3-year follow-up, and the limited number of participating centers, though nationally representative. Our study focused on major organizational changes during the pandemic, attempting to uncover hidden aspects through unobserved variable analysis. We did not assess the prognosis of patients who discontinued consultations due to the pandemic, with uncertain impact on cancer prognosis and COVID-19 exposure. The study’s focus on survival outcomes does not address the psychological impact on cancer patients, though research suggests they demonstrated resilience.^46,47^ The brief study period in April 2020 may not capture the full picture of longer-lasting, regionally varied contingency plans. Our study demonstrates healthcare system resilience for patients accessing care during the pandemic peak but cannot fully address the overall impact on gastrointestinal cancer outcomes. The 13% reduction in consultations suggests that many patients did not access care and may present later with advanced disease. The high proportion of metastatic cases (68%), although compatible with the treatment duration dynamics and workload differences between metastatic and non-metastatic cancer patients, still warrants careful consideration. This limitation in generalizability, likely influenced by screening suspensions and potential selection bias, necessitates a cautious interpretation of our findings. Furthermore, our study’s follow-up period, while adequate for assessing outcomes in metastatic disease with relatively mature and precise estimates, may not fully capture the potential increase in recurrence rates for localized cancers. These initially localized cases could potentially progress to metastatic disease in the years following our study, thereby affecting long-term survival outcomes. As evidenced in Figure 3, the survival estimates for localized cancers show considerable imprecision, which limits our ability to draw firm conclusions about how the pandemic impacted early-stage disease outcomes. Given these limitations, follow-up studies are crucial to examine delayed presentations, long-term recurrence patterns, and provide a more comprehensive view of COVID-19’s enduring impact on cancer care across all disease stages.

While acknowledging the limitations, this study reveals important findings. Notably, most patients who were recommended treatment successfully received standard regimens, despite highly variable regional pandemic responses in Spain’s communities. Adaptations were modest, indicating healthcare resilience. Cross-community cooperative groups likely mitigated the impact, enabling consistent patient care. Uniform patient outcomes across regions thus spotlights the adaptability and robustness of Spain’s healthcare infrastructure during the pandemic. These findings show that coordinated efforts between healthcare providers and administrative systems can minimize the negative impacts of future public health crises on cancer patient management and outcomes. However, it is vital that such strategies extend to all patients, including the most vulnerable, to prevent disease progression during crises.

## Supplementary material

Supplementary material is available at *The Oncologist* online.

oyae300_suppl_Supplementary_Material

oyae300_suppl_Supplementary_Table_S10

oyae300_suppl_Supplementary_Table_S3

oyae300_suppl_Supplementary_Table_S4

oyae300_suppl_Supplementary_Table_S5

oyae300_suppl_Supplementary_Table_S6

oyae300_suppl_Supplementary_Table_S7

oyae300_suppl_Supplementary_Table_S8

oyae300_suppl_Supplementary_Table_S9

oyae300_suppl_Supplementary_Figure_S1

oyae300_suppl_Supplementary_Figure_S2

oyae300_suppl_Supplementary_Table_S2

## Data Availability

The analyses were carried out with the statistical package R v4.3.1, including the survival, spBayesSurv, and SpatialPack libraries. The R code for data analysis is in **Supplementary Table 2**.

## References

[1] Soriano JB , Peláez A, Fernández E, Ancochea J. Impact of the COVID-19 pandemic in mortality due to respiratory diseases: a comparative analysis of 2021 and 2020 vs 2019 in Spain. Med Clin. 2023;161:192-198. https://doi.org/10.1016/j.medcli.2023.04.020 PMC10167373 37394353

[2] Spijker JJA , Trias-Llimós S. Cause-specific mortality in Spain during the pandemic: educational differences and its impact on life expectancy. Eur J Public Health. 2023;33:543-549. https://doi.org/10.1093/eurpub/ckad036 36944099 PMC10234659

[3] Sanitarias C de C de A y E. Actualización no 143. Enfermedad por el coronavirus (COVID-19). 2020.

[4] Pérez-Gómez B , Pastor-Barriuso R, Fernández-de-Larrea N, et al SARS-CoV-2 infection during the first and second pandemic waves in Spain: the ENE–COVID study. Am J Public Health. 2023;113:533-544. https://doi.org/10.2105/AJPH.2023.307233 36893370 PMC10088950

[5] Politi J , Martín-Sánchez M, Mercuriali L, et al; COVID-19 Surveillance Working Group of Barcelona. Epidemiological characteristics and outcomes of COVID-19 cases: mortality inequalities by socio-economic status, Barcelona, Spain, 24 February to 4 May 2020. Euro Surveill. 2021;26:2001138. https://doi.org/10.2807/1560-7917.ES.2021.26.20.2001138 34018483 PMC8138960

[6] Kenyon C. COVID-19 infection fatality rate associated with incidence—a population-level analysis of 19 Spanish autonomous communities. Biology. 2020;9:128. https://doi.org/10.3390/biology9060128 32560071 PMC7345771

[7] Saavedra P , Santana A, Bello L, Pacheco J-M, Sanjuán E. A Bayesian spatio-temporal analysis of mortality rates in Spain: application to the COVID-19 2020 outbreak. Popul. Health Metr. 2021;19:1-10. 34059063 10.1186/s12963-021-00259-y PMC8165954

[8] Fernández-Martínez NF , Ruiz-Montero R, Gómez-Barroso D, et al Socioeconomic differences in COVID-19 infection, hospitalisation and mortality in urban areas in a region in the South of Europe. BMC Public Health. 2022;22:1-10. 36503482 10.1186/s12889-022-14774-6 PMC9742010

[9] Marí-Dell’Olmo M , Gotsens M, Pasarín MI, et al Socioeconomic inequalities in COVID-19 in a European urban area: two waves, two patterns. Int J Environ Res Public Health. 2021;18:1256. https://doi.org/10.3390/ijerph18031256 33573323 PMC7908269

[10] Monge S , Zamalloa PL, Moros MJS, et al Lifting COVID-19 mitigation measures in Spain (May–June 2020). Enferm Infecc Microbiol Clin. 2023;41:11-17. 36619362 10.1016/j.eimc.2021.05.011 PMC9791371

[11] Cura-González D , Polentinos-Castro E, Fontan-Vela M, et al What have we missed because of COVID-19? Missed diagnoses and delayed follow-ups. SESPAS Report 2022. Gac Sanit. 2022;36:S36-S43. 35781146 10.1016/j.gaceta.2022.03.003 PMC9244613

[12] Garcia-Olive I , Seguí F, Guillamet G, et al Impacto de la pandemia por Covid-19 en los diagnósticos respiratorios en el Área Metropolitana Norte de Barcelona (España) Impact of the COVID-19 pandemic on diagnosis of respiratory diseases in the Northern Metropolitan Area in Barcelona (Spain). Med Clin. 2023;160:392-396. 10.1016/j.medcli.2022.11.021 PMC9868362 36822982

[13] Rodríguez-Leor O , Cid-Álvarez B, de Prado AP, et al Impact of COVID-19 on ST-segment elevation myocardial infarction care. The Spanish experience. Rev Esp Cardiol (Engl Ed). 2020;73:994-1002. 32917566 10.1016/j.rec.2020.08.002 PMC7834732

[14] Carriazo S , Aparicio-Madre MI, Tornero-Molina F, et al; REMER Committee. Impact of different COVID-19 waves on kidney replacement therapy epidemiology and mortality: REMER 2020. Nephrol Dial Transplant. 2022;37:2253-2263. https://doi.org/10.1093/ndt/gfac234 35927791 PMC9384646

[15] Garrido-Cantero G , Longo F, Hernández-González J, et al; On Behalf Of The Madrid Cancer Registry Rtmad Investigators. Impact of the COVID-19 Pandemic on Cancer Diagnosis in Madrid (Spain) Based on the RTMAD Tumor Registry (2019–2021). Cancers. 2023;15:1753. https://doi.org/10.3390/cancers15061753 36980640 PMC10046347

[16] Suárez J , Mata E, Guerra A, et al Impact of the COVID‐19 pandemic during Spain’s state of emergency on the diagnosis of colorectal cancer. J Surg Oncol. 2021;123:32-36. https://doi.org/10.1002/jso.26263 33078425

[17] Amador M , Matias-Guiu X, Sancho-Pardo G, et al Impact of the COVID-19 pandemic on the care of cancer patients in Spain. ESMO Open. 2021;6:100157. https://doi.org/10.1016/j.esmoop.2021.100157 34015642 PMC8128716

[18] Ruiz-Medina S , Gil S, Jimenez B, et al Significant decrease in annual cancer diagnoses in Spain during the COVID-19 pandemic: a real-data study. Cancers. 2021;13:3215. https://doi.org/10.3390/cancers13133215 34203185 PMC8267675

[19] Ribes J , Pareja L, Sanz X, et al Cancer diagnosis in Catalonia (Spain) after two years of COVID-19 pandemic: an incomplete recovery. ESMO Open. 2022;7:100486. https://doi.org/10.1016/j.esmoop.2022.100486 35714476 PMC9197337

[20] Rogado J , Pangua C, Serrano-Montero G, et al Covid-19 and lung cancer: a greater fatality rate? Lung Cancer. 2020;146:19-22. https://doi.org/10.1016/j.lungcan.2020.05.034 32505076 PMC7260554

[21] Petrova D , Pollán M, Rodriguez-Barranco M, et al Anticipated help-seeking for cancer symptoms before and after the coronavirus pandemic: results from the Onco-barometer population survey in Spain. Br J Cancer. 2021;124:2017-2025. https://doi.org/10.1038/s41416-021-01382-1 33854210 PMC8044659

[22] Galipienzo J , Otta-Oshiro RJ, Salvatierra D, et al Perioperative management of non-deferrable oncologic surgeries during COVID-19 pandemic in Madrid, Spain. Is it safe? Rev Esp Anestesiol Reanim. 2022;69:25-33. https://doi.org/10.1016/j.redare.2021.03.005 35033483 PMC8754582

[23] Martínez-Hernández NJ , Caballero Silva U, Cabañero Sánchez A, et al; On Behalf Of The Scientific Committee Of The Spanish Thoracic Surgery Society. Effect of COVID-19 on thoracic oncology surgery in Spain: a Spanish Thoracic Surgery Society (SECT) survey. Cancers 2021;13:2897. https://doi.org/10.3390/cancers13122897 34207878 PMC8226458

[24] Ruda‐Santolaria L , Bergerot C, Hernandez J, et al Use of telehealth for psychosocial oncology: a mixed methods study about barriers to and opportunities with Latino patients from Latin America, Spain, and the United States. Psychosoc. Oncol. 2023;32:1289-1297. https://doi.org/10.1002/pon.6182 PMC11289857 37370195

[25] Curigliano G , Banerjee S, Cervantes A, et al; Panel members. Managing cancer patients during the COVID-19 pandemic: an ESMO multidisciplinary expert consensus. Ann. Oncol. 2020;31:1320-1335. https://doi.org/10.1016/j.annonc.2020.07.010 32745693 PMC7836806

[26] Vecchione L , Stintzing S, Pentheroudakis G, Douillard J-Y, Lordick F. ESMO management and treatment adapted recommendations in the COVID-19 era: colorectal cancer. ESMO Open. 2020;5:e000826. https://doi.org/10.1136/esmoopen-2020-000826 32457036 PMC7276236

[27] Médica SSE de O. Recomendaciones sobre Covid-19. Accessed October 25, 2024. https://seom.org/recomendaciones-sobre-covid-19

[28] Cooner F , Banerjee S, McBean AM. Modelling geographically referenced survival data with a cure fraction. Stat Methods Med Res. 2006;15:307-324. https://doi.org/10.1191/0962280206sm453oa 16886733 PMC2963459

[29] Hougaard P. Frailty models for survival data. Lifetime Data Anal. 1995;1:255-273. https://doi.org/10.1007/BF00985760 9385105

[30] Zhou H , Hanson T, Zhang J. spBayesSurv: Bayesian Modeling and Analysis of Spatially Correlated Survival Data. Version 1.1.8. 2024. Accessed November 1, 2024. https://cran.r-project.org/web/packages/spBayesSurv/spBayesSurv.pdf

[31] Vallejos R , Osorio F, Bevilacqua M. Tjøstheim’s Coefficient. In: Spatial Relationships Between Two Georeferenced Variables: With Applications in R. Cham: Springer; 2020. p. 69–78. Accessed November 1, 2024. https://link.springer.com/chapter/10.1007/978-3-030-56681-4_4

[32] R Core Team. R: a language and environment for statistical computing. R Foundation for Statistical Computing; 2014.

[33] Osorio F , Vallejos R, Cuevas F. SpatialPack: Computing the association between two spatial processes. arXiv preprint. 2016. Accessed November 1, 2024. https://arxiv.org/abs/1611.05289

[34] Therneau TM , Lumley T. Package “survival.” Version 3.7-0. 2024. Accessed November 1, 2024. https://cran.r-project.org/package=survival

[35] Condes E , Arribas JR; COVID19 MADRID-S.P.P.M. group. Impact of COVID-19 on Madrid hospital system. Enferm Infecc Microbiol Clin. 2021;39:256-257. https://doi.org/10.1016/j.eimc.2020.06.005 38620683 PMC7315960

[36] Gallego-Plazas J , Arias-Martinez A, Arrazubi V. Sex and gender disparities in patients with advanced gastroesophageal adenocarcinoma: data from AGAMENON-SEOM registry. ESMO Open. 2022;7:100514. 35714478 10.1016/j.esmoop.2022.100514 PMC9271495

[37] Contreras-Toledo D , Jiménez-Fonseca P, López CL, et al Dynamic nature of BRAF or KRAS p.G12C mutations in second-line therapy for advanced colorectal cancer patients: do early and late effects exist? Br J Cancer. 2024;130:777-787. https://doi.org/10.1038/s41416-023-02563-w 38191609 PMC10912758

[38] Fernández A , Salgado M, García A, et al Prognostic factors for survival with nab-paclitaxel plus gemcitabine in metastatic pancreatic cancer in real-life practice: the ANICE-PaC study. BMC Cancer. 2018;18:1185. https://doi.org/10.1186/s12885-018-5101-3 30497432 PMC6267080

[39] López J , Jiménez-Fonseca P, Fernández-Montes A. Treatment patterns and outcomes in localized esophagogastric cancer according to histology and location: data from the national AGAMENON-SEOM registry. In: Abstract Book of the Annual Meeting of the Spanish Society of Medical Oncology (SEOM); 2023.

[40] Legido-Quigley H , Mateos-García JT, Campos VR, et al The resilience of the Spanish health system against the COVID-19 pandemic. The Lancet Public Health. 2020;5:e251-e252. https://doi.org/10.1016/S2468-2667(20)30060-8 32199083 PMC7104264

[41] Sánchez-Bayón A , González-Arnedo E, Andreu-Escario A. Spanish healthcare sector management in the COVID-19 crisis under the perspective of Austrian economics and new-institutional economics. Front Public Health. 2022;10:801525. https://doi.org/10.3389/fpubh.2022.801525 35372254 PMC8971546

[42] Lopez-Villegas A , Bautista-Mesa RJ, Baena-Lopez MA, et al Impact of the COVID-19 pandemic on healthcare activity in the regional hospitals of Andalusia (Spain). J. Clin. Med. 2022;11:363. https://doi.org/10.3390/jcm11020363 35054055 PMC8781610

[43] Nuñez JH , Porcel JA, Pijoan J, et al Rethinking Trauma Hospital Services in one of Spain’s Largest University Hospitals during the COVID-19 pandemic. How can we organize and help? Our experience. Injury. 2020;51:2827-2833. https://doi.org/10.1016/j.injury.2020.09.055 33004206 PMC7518794

[44] Lopez-Picazo JJ , Vidal-Abarca I, Beteta D, López-Ibáñez M, García-Vázquez E. Impact of the COVID-19 pandemic on the hospital: inpatient’s perceived quality in Spain. J. Patient Exp. 2021;8:2374373521998625. https://doi.org/10.1177/2374373521998625 34179398 PMC8205378

[45] Westreich D. Berkson’s bias, selection bias, and missing data. Epidemiology. 2012;23:159-164. https://doi.org/10.1097/EDE.0b013e31823b6296 22081062 PMC3237868

[46] Obispo-Portero B , Cruz-Castellanos P, Jiménez-Fonseca P, et al Anxiety and depression in patients with advanced cancer during the COVID-19 pandemic. Support Care Cancer. 2022;30:3363-3370. https://doi.org/10.1007/s00520-021-06789-3 34993652 PMC8735888

[47] Velasco-Durantez V , Jimenez-Fonseca P, Martín Abreu CM, et al Resilience, social support, and anxious preoccupation in patients with advanced cancer during COVID-19 pandemic. Cancer Invest. 2022;40:475-482. https://doi.org/10.1080/07357907.2022.2067864 35468046

