# Supplementary material for: Three-year survival follow-up of patients with gastrointestinal cancer treated during the COVID-19 pandemic in Spain: data from the PANDORA-TTD20 study
Source: Oncologist. 2024 Nov 16;30(8):oyae300. doi: 10.1093/oncolo/oyae300 (PMC12395236; doi:10.1093/oncolo/oyae300)
Supplement: oyae300_suppl_Supplementary_Material [file oyae300_suppl_supplementary_material.docx]

**Methods**

**Study design and population**

Data for this research were sourced from the Spanish Registry of Digestive Tumors (RETUD), an observational, multicenter epidemiological study on gastrointestinal tumors supported by TTD. Within this registry, PANDORA-TTD20 was established as a retrospective cohort, involving 19 TTD-affiliated centers situated in regions that collectively encompass approximately 72% of Spain's population. All participating centers are providers of specialized tertiary cancer care in Spain, with further details available in **Supplementary Table 1**.

The study population consisted of patients seen in consultation between April 20 and 24, 2020, the peak of Spain's first COVID-19 wave when pandemic-related disruptions were most severe. Eligibility criteria included patients who were over 18 years old, diagnosed with gastrointestinal cancer, and treated at the Oncology Department, regardless of tumor stage or antineoplastic treatment status. Outcome analyses were performed on patients with localized tumors under active treatment (excluding follow-up consultations) as well as all patients with advanced disease stages. Follow-up in April 2023 enabled 3-year survival assessment.

An additional survey was administered to the centers to investigate staffing within the medical oncology services, activity of the digestive tumor committees, and the number of consecutive patients seen in the digestive tumor consultations of the medical oncology department before (February 2020) and during the peak of the first wave (April 2020).

All alive participants at the time of recruitment provided informed consent. This study was conducted in accordance with the ethical regulations of each participating center, ensuring the protection of rights and safety of patients in accordance with local laws and international ethical principles.

**Endpoints and variables**

The primary aim was to examine the changes in organization, structures, staffing, and committee that could have affected the management and outcomes of patients with digestive cancer during the peak of Spain's COVID-19 crisis. Part of this goal was to identify any potential spatial frailties that might be associated with regional disparities. Such disparities could stem from unmeasured factors not accounted for by the variables included in this study, and may merit further detailed investigation ^1^. The endpoint selected for the analysis of these potential vulnerabilities was overall survival (OS), ascertained from April 22, 2020, until any-cause death, censoring subjects without an event at the final follow-up point (April 20, 2023). This date was chosen to model data emerging from the pandemic's peak, considering tumor diversity, stages, and oncological circumstances.

The secondary aims were to gain insight into cancer management patterns during the pandemic across various centers.

Medical variables such as Eastern Cooperative Oncology Group performance status (ECOG-PS), comorbidities, age, sex, disease stage, primary tumor location, systemic antineoplasic treatment goals, and participation in a clinical trial, were extracted from patient's medical records. These covariates were selected theoretically after a review of the literature and consensus among the project coordinators. Further explanatory variables included localized and metastatic disease management patterns, dose and interval adjustments and omissions from the treatment regimen, consultation visit adaptations, SARS-CoV-2 infection rates, and the direct consequences of the infection on patients.

An additional survey was administered to the centers to investigate staffing within the medical oncology services, activity of the digestive tumor committees, and the number of consecutive patients seen in the digestive tumor consultations of the medical oncology department before (February 2020) and during the peak of the first wave (April 2020). The lead oncologists in charge of digestive tumors at each hospital completed this questionnaire.

The data on the COVID-19 situation in each autonomous community were obtained from the Ministry of Health of Spain ^2^.

**Statistical method**

The study took into account spatial location as it plays a role in predicting survival outcomes as a proxy for unmeasured regional characteristics that may have influenced the evolution of COVID-19 in these patients. For this purpose, we fitted a Bayesian semiparametric proportional hazards (PH) model, where different regional locations were modeled via frailties ^1,3^. This model applied an independent identically distributed (IID) non-informative Gaussian prior, assuming that regional lockdowns in Spain implied non-spatial data. As an additional analysis, a similar model was fitted assuming a Gaussian random field (GRF) prior that consider the amount of spatial variation between specific centers, based on their geographic coordinates ^4^. The aim of this second analysis was to try to capture possible proximity relationships between centers belonging to the same city, without assuming complete regional representativeness. In both cases, a multivariable model was specified with all the previously mentioned covariates. The exponentiated frailties (adjusted hazard rates) were represented on a geographic map, assuming the regional representativeness of each center. The correlation between regional frailties and the actual situation of COVID-19 was quantified using Tjostheim’s coefficient for two spatial sequences observed over the same locations on the plane. Tjostheim’s coefficient is a variant of the correlation coefficient to be used in a spatial statistics context ^5^. Basic summary statistics, including medians and percentages, were utilized for the analysis of the remaining descriptive variables. The Kaplan-Meier estimator was employed to model survival. A two-tailed Wilcoxon test for paired data was implemented to compare longitudinal counts from the periods before and during the pandemic. The analyses were carried out with the statistical package R v4.3.1 ^6^, including the survival, spBayesSurv and SpatialPack libraries ^4,7,8^. The R code for data analysis is in **Supplementary Table 2**.

**Bibliography**

1 Cooner F, Banerjee S, McBean AM. Modelling geographically referenced survival data with a cure fraction. Stat Methods Med Res 2006;15:307–324.

2 Sanitarias C de C de A y E. Actualización no 143. Enfermedad por el coronavirus (COVID-19). 21.06.2020. Madrid.

3 Hougaard P. Frailty models for survival data. Lifetime Data Anal 1995;1:255–273.

4 Zhou H, Hanson T, Zhang J. spBayesSurv: Fitting Bayesian spatial survival models using R. arXiv preprint arXiv:170504584 2017.

5 Vallejos R, Osorio F, Bevilacqua M et al. Tjøstheim’s Coefficient. Spatial Relationships Between Two Georeferenced Variables: With Applications in R 2020;:69–78.

6 R Core Team. R: a language and environment for statistical computing. Vienna, Austria: R Foundation for Statistical Computing. 2014.

7 Osorio F, Vallejos R, Cuevas F. SpatialPack: Computing the association between two spatial processes. arXiv preprint arXiv:161105289 2016.

8 Therneau TM, Lumley T. Package ‘survival.’ 2016.Available at https://cran.r-project.org/package=survival.
