# Supplementary material for: Three-year survival follow-up of patients with gastrointestinal cancer treated during the COVID-19 pandemic in Spain: data from the PANDORA-TTD20 study
Source: Oncologist. 2024 Nov 16;30(8):oyae300. doi: 10.1093/oncolo/oyae300 (PMC12395236; doi:10.1093/oncolo/oyae300)
Supplement: oyae300_suppl_Supplementary_Table_S10 [file oyae300_suppl_supplementary_table_s10.docx]

**Supplementary Table 10**. The model for overall survival with non-frailty variables.

| **Variable** | **Mean** | **Median** | **Std. Dev.** | **95%CI-Low** | **95%CI-Upper** | **HR** | **LL95%** | **UL95%** |
| --- | --- | --- | --- | --- | --- | --- | --- | --- |
| **Systemic treatment**  Adjuvant Therapy (ref.)  First line  Second line  Third or subsequent lines | -  -0.28  -0.60  -0.17 | -  -0.29  -0.61  -0.15 | -  0.12  0.24  0.15 | -  -0.50  -1.04  -0.49 | -  -0.06  -0.06  0.08 | -  0.75  0.55  0.84 | -  0.60  0.35  0.61 | -  0.94  0.94  1.08 |
| **Primary cancer site**  Esophagus (ref.)  Stomach  Pancreas  Hepatobiliary  Colon  Rectum  Anus | -  -0.13  0.10  0.03  -0.83  -0.39  -0.32 | -  -0.14  0.10  0.02  -0.84  -0.38  -0.27 | -  0.27  0.22  0.23  0.22  0.24  0.56 | -  -0.62  -0.28  -0.39  -1.23  -0.86  -1.52 | -  0.38  0.51  0.50  -0.45  0.06  0.69 | -  0.88  1.11  1.03  0.43  0.68  0.73 | -  0.54  0.75  0.68  0.29  0.42  0.22 | -  1.46  1.67  1.66  0.63  1.06  1.99 |
| **Stage**  Non-metastatic (ref.)  Metastatic | -  1.51 | -  1.52 | -  0.18 | -  1.20 | -  1.87 | -  4.54 | -  3.32 | -  6.47 |
| **ECOG-PS**  0 (ref.)  1  2  3-4  Not available | -  0.74  1.61  2.34  0.79 | -  0.74  1.62  2.34  0.78 | -  0.11  0.19  0.26  0.18 | -  0.51  1.27  1.82  0.45 | -  0.97  1.94  2.85  1.15 | -  2.10  5.02  10.41  2.20 | -  1.67  3.55  6.17  1.57 | -  2.63  6.99  17.37  3.15 |

**Abbreviations**: Ref., reference; Std. Dev., Standard Deviation; CI, Confidence Interval; HR, hazard Ratio; LL, Low Limit; UL, Upper Limit; ECOG-PS, Eastern Cooperative Oncology Group - Performance Status.
