# Supplementary material for: Three-year survival follow-up of patients with gastrointestinal cancer treated during the COVID-19 pandemic in Spain: data from the PANDORA-TTD20 study
Source: Oncologist. 2024 Nov 16;30(8):oyae300. doi: 10.1093/oncolo/oyae300 (PMC12395236; doi:10.1093/oncolo/oyae300)
Supplement: oyae300_suppl_Supplementary_Table_S3 [file oyae300_suppl_supplementary_table_s3.docx]

**Supplementary Table 3**. Characteristics of participating medical oncology departments (n=19).

| **Characteristics of participating medical oncology departments** | **Data** |
| --- | --- |
| **Population covered by center, number (%)**  **100.000-200000**  **200001-300000**  **>300000** | 1 (5.2)  1 (5.2)  17 (89.4) |
| **Data Out of Pandemic Period (February 2020)** |  |
| **Oncologists (total, specialists, and resident medical interns) in the department, median (range)** | 28 (16-65) |
| **Oncologists (specialists) in the department, median (range)** | 15 (5-50) |
| **Oncologists (specialists) in the gastrointestinal tumors section, median (range)** | 5 (2-12) |
| **Oncologists on sick leave (specialists and resident medical interns) in the department, median (range)** | 0 (0-3) |
| **Data During Pandemic Period (April 2020)** |  |
| **Oncologists on sick leave (specialists and resident medical interns) in the department, median (range)** | 4 (0-11) |
| **Oncologists on sick leave due to COVID-19, median (range)** | 2 (0-9) |
| **Oncologist suffering COVID, median (range)** | 1 (0-9) |
| **Quarantine, median (range)** | 0 (0-5) |
| **The department adapted to the pandemic situation, number (%)**  **maintaining healthcare activity with minimal changes**  **adapting the workforce to shift work**  **by suspending almost all activities** | 7 (36.8)  11 (57.8)  1 (5.2) |
| **Guidelines followed in the gastrointestinal tumor section for therapeutic adjustment during the pandemic, number (%)**  **SEOM guidelines and/or SEOM-GI Tumor Treatment**  **ESMO guidelines**  **Departmental guidelines or consensus**  **Decisions were based on the individual oncologist's criteria** | 11 (57.8)  3 (15.7)  5 (26.3)  0 |
