# Supplementary material for: Three-year survival follow-up of patients with gastrointestinal cancer treated during the COVID-19 pandemic in Spain: data from the PANDORA-TTD20 study
Source: Oncologist. 2024 Nov 16;30(8):oyae300. doi: 10.1093/oncolo/oyae300 (PMC12395236; doi:10.1093/oncolo/oyae300)
Supplement: oyae300_suppl_Supplementary_Table_S4 [file oyae300_suppl_supplementary_table_s4.docx]

| **Variable** | **Total**  **N (%)** | **CHUA** | **CHUO** | **HUCA** | **HUMV** | **HU Donostia** | **HUN** | **HUMS** | **VHIO** | **ICO** | **H. San Pau** | **HGU de Valencia** | **IVO** | **HGU de Elche** | **HGUGM** | **HU La Paz** | **HURC** | **HURS** | **HUVR** | **H. Regional Univ Málaga** |
| --- | --- | --- | --- | --- | --- | --- | --- | --- | --- | --- | --- | --- | --- | --- | --- | --- | --- | --- | --- | --- |
| **Total patients per center** | **703 (100)** | **24 (100)** | **15 (100)** | **47 (100)** | **14 (100)** | **69 (100)** | **32 (100)** | **37 (100)** | **71 (100)** | **57 (100)** | **27 (100)** | **21 (100)** | **32 (100)** | **19 (100)** | **78 (100)** | **36 (100)** | **10 (100)** | **51 (100)** | **37 (100)** | **26 (100)** |
| **Age, mean (range)** | 66.2  (30 - 89.3) | 62.5  (32.5 - 80.7) | 63.1  (47.5 - 82.1) | 61.4  (30 - 80.9) | 73.7  (58.8 - 86.4) | 66.4  (40.3 - 87.4) | 66.8  (40.3 - 84.9) | 66.2  (36.4 - 86.1) | 65.7  (34.6 - 85.3) | 66.6  (42 - 87.3) | 70.7  (51 - 86.1) | 69.9  (43.4 - 84.8) | 63.3  (45.4 - 79.6) | 60.9  (43.3 - 80.3) | 64.8  (31.8 - 89.3) | 65.7  (44.5 - 84.3) | 63.4  (41.7 - 82.2) | 71.1  (49.8 - 84) | 65  (34.5 - 83.5) | 68.9  (45 - 84.4) |
| **Sex, women** | 260 (36.98) | 9 (37.5) | 6 (40) | 20 (42.55) | 2 (14.29) | 23 (33.33) | 13 (40.62) | 16 (43.24) | 30 (42.25) | 20 (35.09) | 13 (48.15) | 12 (57.14) | 8 (25) | 7 (36.84) | 24 (30.77) | 15 (41.67) | 5 (50) | 13 (25.49) | 14 (37.84) | 10 (38.46) |
| **ECOG-PS**  **0**  **1**  **2**  **3**  **4**  **Unknown** | 209 (29.73)  341 (48.51)  66 (9.39)  16 (2.28)  5 (0.71)  66 (9.39) | 0 (0)  13 (54.17)  7 (29.17)  2 (8.33)  2 (8.33)  0 (0) | 5 (33.33)  9 (60)  1 (6.67) | 14 (29.79)  28 (59.57)  5 (10.64)  0 (0)  0 (0)  0 (0) | 6 (42.86)  3 (21.43)  2 (14.29)  1 (7.14)  0 (0)  2 (14.29) | 34 (49.28)  25 (36.23)  10 (14.49)  0 (0)  0 (0)  0 (0) | 2 (6.25)  27 (84.38)  3 (9.38)  0 (0)  0 (0)  0 (0) | 10 (27.03)  5 (13.51)  4 (10.81)  1 (2.7)  1 (2.7)  16 (43.24) | 13 (18.31)  55 (77.46)  3 (4.23)  0 (0)  0 (0)  0 (0) | 21 (36.84)  28 (49.12)  4 (7.02)  0 (0)  0 (0)  4 (7.02) | 7 (25.93)  14 (51.85)  1 (3.7)  1 (3.7)  0 (0)  4 (14.81) | 12 (57.14)  1 (4.76)  0 (0)  0 (0)  0 (0)  8 (38.1) | 11 (34.38)  17 (53.12)  4 (12.5)  0 (0)  0 (0)  0 (0) | 3 (15.79)  12 (63.16)  3 (15.79)  1 (5.26)  0 (0)  0 (0) | 28 (35.9)  34 (43.59)  4 (5.13)  6 (7.69)  0 (0)  6 (7.69) | 7 (19.44)  12 (33.33)  4 (11.11)  1 (2.78)  0 (0)  12 (33.33) | 2 (20)  4 (40)  2 (20)  1 (10)  1 (10)  0 (0) | 18 (35.29)  29 (56.86)  3 (5.88)  1 (1.96)  0 (0)  0 (0) | 1 (2.7)  14 (37.84)  6 (16.22)  1 (2.7)  1 (2.7)  14 (37.84) | 15 (57.69)  11 (42.31)  0 (0)  0 (0)  0 (0)  0 (0) |
| **Comorbidities that limit systemic treatment*** | 128 (18.21) | 7 (29.17) | 7 (46.67) | 4 (8.51) | 0 (0) | 15 (21.74) | 9 (28.12) | 0 (0) | 11 (15.49) | 30 (52.63) | 4 (14.81) | 1 (4.76) | 5 (15.62) | 3 (15.79) | 21 (26.92) | 0 (0) | 3 (30) | 0 (0) | 3 (8.11) | 5 (19.23) |
| **Patient referred from another area or health center** | 138 (19.63) | 1 (4.17) | 1 (6.67) | 1 (2.13) | 3 (21.43) | 8 (11.59) | 0 (0) | 4 (10.81) | 35 (49.3) | 50 (87.72) | 2 (7.41) | 0 (0) | 1 (3.12) | 1 (5.26) | 12 (15.38) | 1 (2.78) | 2 (20) | 14 (27.45) | 2 (5.41) | 0 (0) |
| **Primary cancer site**  **Esophagus**  **Stomach**  **Pancreas**  **Liver and bile duct**  **Colon**  **Rectum**  **Anus** | 41 (5.83)  47 (6.69)  150 (21.34)  64 (9.1)  266 (37.84)  127 (18.07)  8 (1.14) | 2 (8.33)  3 (12.5)  4 (16.67)  4 (16.67)  9 (37.5)  1 (4.17)  1 (4.17) | 0 (0)  2 (13.33)  5 (33.33)  1 (6.67)  5 (33.33)  2 (13.33)  0 (0) | 3 (6.38)  7 (14.89)  9 (19.15)  3 (6.38)  22 (46.81)  3 (6.38)  0 (0) | 0 (0)  1 (7.14)  2 (14.29)  3 (21.43)  5 (35.71)  3 (21.43)  0 (0) | 3 (4.35)  6 (8.7)  17 (24.64)  12 (17.39)  23 (33.33)  8 (11.59)  0 (0) | 2 (6.25)  1 (3.12)  5 (15.62)  1 (3.12)  15 (46.88)  8 (25)  0 (0) | 6 (16.22)  0 (0)  9 (24.32)  2 (5.41)  11 (29.73)  7 (18.92)  2 (5.41) | 0 (0)  3 (4.23)  23 (32.39)  14 (19.72)  19 (26.76)  11 (15.49)  1 (1.41) | 0 (0)  0 (0)  11 (19.3)  4 (7.02)  23 (40.35)  19 (33.33)  0 (0) | 2 (7.41)  3 (11.11)  7 (25.93)  2 (7.41)  9 (33.33)  4 (14.81)  0 (0) | 0 (0)  2 (9.52)  1 (4.76)  0 (0)  13 (61.9)  5 (23.81)  0 (0) | 3 (9.38)  2 (6.25)  5 (15.62)  3 (9.38)  10 (31.25)  8 (25)  1 (3.12) | 0 (0)  3 (15.79)  5 (26.32)  0 (0)  6 (31.58)  5 (26.32)  0 (0) | 10 (12.82)  6 (7.69)  11 (14.1)  9 (11.54)  34 (43.59)  8 (10.26)  0 (0) | 2 (5.56)  1 (2.78)  19 (52.78)  2 (5.56)  9 (25)  2 (5.56)  1 (2.78) | 0 (0)  1 (10)  1 (10)  0 (0)  5 (50)  2 (20)  1 (10) | 2 (3.92)  4 (7.84)  6 (11.76)  3 (5.88)  21 (41.18)  15 (29.41)  0 (0) | 6 (16.22)  2 (5.41)  10 (27.03)  1 (2.7)  11 (29.73)  6 (16.22)  1 (2.7) | 0 (0)  0 (0)  0 (0)  0 (0)  16 (61.54)  10 (38.46)  0 (0) |
| **Tumor stage**  **Non metastatic**  **Metastatic** | 221 (31.44)  482 (68.56) | 0 (0)  24 (100) | 3 (20)  12 (80) | 10 (21.28)  37 (78.72) | 4 (28.57)  10 (71.43) | 23 (33.33)  46 (66.67) | 15 (46.88)  17 (53.12) | 7 (18.92)  30 (81.08) | 15 (21.13)  56 (78.87) | 32 (56.14)  25 (43.86) | 15 (55.56)  12 (44.44) | 14 (66.67)  7 (33.33) | 5 (15.62)  27 (84.38) | 6 (31.58)  13 (68.42) | 15 (19.23)  63 (80.77) | 9 (25)  27 (75) | 0 (0)  10 (100) | 23 (45.1)  28 (54.9) | 15 (40.54)  22 (59.46) | 10 (38.46)  16 (61.54) |
| **Clinical trial participants** | 76 (10.81) | 2 (8.33) | 1 (6.67) | 8 (17.02) | 0 (0) | 2 (2.9) | 0 (0) | 13 (35.14) | 23 (32.39) | 2 (3.51) | 3 (11.11) | 0 (0) | 0 (0) | 2 (10.53) | 13 (16.67) | 4 (11.11) | 0 (0) | 0 (0) | 1 (2.7) | 2 (7.69) |

**Supplementary Table 4.** Baseline characteristics by center.

**Abbreviations**: CHUA, Complejo Hospitalario Universitario de A Coruña; CHUO, Complejo Hospitalario Universitario de Orense; HUCA, Hospital Universitario Central de Asturias, HUMV, Hospital Universitario Marqués de Valdecilla; HUN, Hospital Universitario de Navarra; HUMS, Hospital Universitario Miguel Servet; VHIO, Hospital Universitario de la Vall d'Hebron y Vall d'Hebron Instituto de Oncología; ICO, Instituto Catalán de Oncología; H. San Pau, Hospital de la Santa Creu i Sant Pau; HGU de Valencia, Hospital General Universitario de Valencia; IVO, Instituto Valenciano de Oncología; HGU de Elche, Hospital General Universitario de Elche; HGUGM, Hospital General Universitario Gregorio Marañón; HU La Paz, Hospital Universitario La Paz; HURC, Hospital Universitario Ramón y Cajal; HURS, Hospital Universitario Reina Sofía; HUVR, Hospital Universitario Virgen del Rocío. Note: * Comorbidities in April 2020 that were deemed sufficiently serious by the oncologist to prevent the initiation or continuation of oncological treatment in patients with indications at the time of evaluation.
