# Supplementary material for: Three-year survival follow-up of patients with gastrointestinal cancer treated during the COVID-19 pandemic in Spain: data from the PANDORA-TTD20 study
Source: Oncologist. 2024 Nov 16;30(8):oyae300. doi: 10.1093/oncolo/oyae300 (PMC12395236; doi:10.1093/oncolo/oyae300)
Supplement: oyae300_suppl_Supplementary_Table_S5 [file oyae300_suppl_supplementary_table_s5.docx]

**Supplementary Table 5.** Management of metastatic cancer patients by center during the COVID-19 pandemic.

| **Variable** | **Total**  **N (%)** | **CHUA** | **CHUO** | **HUCA** | **HUMV** | **HU Donostia** | **HUN** | **HUMS** | **VHIO** | **ICO** | **H San Pau** | **HGU de Valencia** | **HGU de Elche** | **HGUGM** | **HU La Paz** | **HURC** | **HURS** | **HUVR** | **IVO** | **H Regional Univ Málaga** |
| --- | --- | --- | --- | --- | --- | --- | --- | --- | --- | --- | --- | --- | --- | --- | --- | --- | --- | --- | --- | --- |
| **Total patients with metastatic cancer per center** | 482 (100) | 24 (100) | 12 (100) | 37 (100) | 10 (100) | 46 (100) | 17 (100) | 30 (100) | 56 (100) | 25 (100) | 12 (100) | 7 (100) | 13 (100) | 63 (100) | 27 (100) | 10 (100) | 28 (100) | 22 (100) | 27 (100) | 16 (100) |
| **Follow-up visit** | 109 (22.61) | 18 (75) | 0 (0) | 0 (0) | 4 (40) | 8 (17.39) | 3 (17.65) | 2 (6.67) | 3 (5.36) | 14 (56) | 2 (16.67) | 5 (71.43) | 2 (15.38) | 5 (7.94) | 6 (22.22) | 2 (20) | 9 (32.14) | 21 (95.45) | 1 (3.7) | 4 (25) |
| **Systemic treatment prescribed despite the pandemic** | 272 (56.43) | 4 (16.67) | 11 (91.67) | 16 (43.24) | 5 (50) | 34 (73.91) | 12 (70.59) | 10 (33.33) | 27 (48.21) | 10 (40) | 8 (66.67) | 1 (14.29) | 10 (76.92) | 46 (73.02) | 19 (70.37) | 7 (70) | 18 (64.29) | 0 (0) | 24 (88.89) | 10 (62.5) |
| **Reasons for not initiating or continuing systemic treatment**  **Improved supportive care as systemic treatment is contraindicated**  **Improved supportive care favored by the pandemic**  **Mixed reasons** | 10 (2.07)  7 (1.45)  24 (4.98) | 0 (0)  0 (0)  0 (0) | 0 (0)  0 (0)  0 (0) | 1 (2.7)  4 (10.81)  8 (21.62) | 0 (0)  0 (0)  1 (10) | 0 (0)  1 (2.17)  1 (2.17) | 1 (5.88)  0 (0)  1 (5.88) | 1 (3.33)  0 (0)  5 (16.67) | 4 (7.14)  0 (0)  2 (3.57) | 0 (0)  0 (0)  0 (0) | 0 (0)  0 (0)  0 (0) | 0 (0)  1 (14.29)  0 (0) | 0 (0)  0 (0)  0 (0) | 2 (3.17)  0 (0)  3 (4.76) | 0 (0)  0 (0)  1 (3.7) | 0 (0)  0 (0)  1 (10) | 0 (0)  1 (3.57)  0 (0) | 0 (0)  0 (0)  0 (0) | 1 (3.7)  0 (0)  1 (3.7) | 0 (0)  0 (0)  0 (0) |
| **Type of systemic treatment**  **First-line chemotherapy with palliative intention**  **First-line chemotherapy with intention to convert**  **Second-line chemotherapy**  **Third-line chemotherapy or beyond**  **Adjuvant chemotherapy for metastasis resection** | 126 (26.14)  40 (8.3)  67 (13.9)  31 (6.43)  8 (1.66) | 4 (16.67)  0 (0)  0 (0)  0 (0)  0 (0) | 7 (58.33)  2 (16.67)  2 (16.67)  0 (0)  0 (0) | 10 (27.03)  2 (5.41)  3 (8.11)  1 (2.7)  0 (0) | 3 (30)  0 (0)  1 (10)  1 (10)  0 (0) | 17 (36.96)  4 (8.7)  9 (19.57)  3 (6.52)  1 (2.17) | 7 (41.18)  1 (5.88)  4 (23.53)  0 (0)  0 (0) | 5 (16.67)  0 (0)  4 (13.33)  1 (3.33)  0 (0) | 10 (17.86)  0 (0)  9 (16.07)  8 (14.29)  0 (0) | 5 (20)  1 (4)  2 (8)  2 (8)  0 (0) | 3 (25)  1 (8.33)  3 (25)  1 (8.33)  0 (0) | 1 (14.29)  0 (0)  0 (0)  0 (0)  0 (0) | 5 (38.46)  0 (0)  2 (15.38)  3 (23.08)  0 (0) | 17 (26.98)  9 (14.29)  14 (22.22)  2 (3.17)  4 (6.35) | 3 (11.11)  9 (33.33)  2 (7.41)  3 (11.11)  2 (7.41) | 5 (50)  0 (0)  1 (10)  1 (10)  0 (0) | 8 (28.57)  7 (25)  3 (10.71)  0 (0)  0 (0) | 0 (0)  0 (0)  0 (0)  0 (0)  0 (0) | 13 (48.15)  1 (3.7)  8 (29.63)  2 (7.41)  0 (0) | 3 (18.75)  3 (18.75)  0 (0)  3 (18.75)  1 (6.25) |
| **Modification of the regimen**  **Alternative antineoplastic agents to the standard**  **Standard regimen adjusting dose**  **Standard regimen adjusting interval**  **Standard regimen adjusting dose and interval**  **Change the route of administration of any drug from IV to oral** | 50 (10.37)  9 (1.87)  15 (3.11)  17 (3.53)  6 (1.24)  3 (0.62) | 2 (8.33)  0 (0)  0 (0)  0 (0)  1 (4.17)  1 (4.17) | 2 (16.67)  1 (8.33)  0 (0)  1 (8.33)  0 (0)  0 (0) | 9 (24.32)  4 (10.81)  2 (5.41)  2 (5.41)  0 (0)  1 (2.7) | 1 (10)  1 (10)  0 (0)  0 (0)  0 (0)  0 (0) | 4 (8.7)  0 (0)  3 (6.52)  0 (0)  1 (2.17)  0 (0) | 6 (35.29)  0 (0)  0 (0)  5 (29.41)  1 (5.88)  0 (0) | 0 (0)  0 (0)  0 (0)  0 (0)  0 (0)  0 (0) | 0 (0)  0 (0)  0 (0)  0 (0)  0 (0)  0 (0) | 1 (4)  0 (0)  0 (0)  0 (0)  1 (4)  0 (0) | 1 (8.33)  0 (0)  0 (0)  0 (0)  0 (0)  1 (8.33) | 0 (0)  0 (0)  0 (0)  0 (0)  0 (0)  0 (0) | 2 (15.38)  0 (0)  0 (0)  2 (15.38)  0 (0)  0 (0) | 11 (17.46)  1 (1.59)  3 (4.76)  5 (7.94)  2 (3.17)  0 (0) | 1 (3.7)  1 (3.7)  0 (0)  0 (0)  0 (0)  0 (0) | 1 (10)  0 (0)  1 (10)  0 (0)  0 (0)  0 (0) | 5 (17.86)  1 (3.57)  2 (7.14)  2 (7.14)  0 (0)  0 (0) | 0 (0)  0 (0)  0 (0)  0 (0)  0 (0)  0 (0) | 4 (14.81)  0 (0)  4 (14.81)  0 (0)  0 (0)  0 (0) | 0 (0)  0 (0)  0 (0)  0 (0)  0 (0)  0 (0) |
| **Modification of visits**  **Several cycles were scheduled without visits and without blood tests**  **In-person and telephone visits were alternated**  **Only telephone visits were conducted**  **Visits were spaced out** | 51 (10.58)  3 (0.62)  31 (6.43)  3 (0.62)  16 (3.32) | 0 (0)  0 (0)  0 (0)  0 (0)  0 (0) | 2 (16.67)  1 (8.33)  0 (0)  0 (0)  1 (8.33) | 8 (21.62)  2 (5.41)  4 (10.81)  0 (0)  2 (5.41) | 0 (0)  0 (0)  0 (0)  0 (0)  0 (0) | 3 (6.52)  0 (0)  3 (6.52)  0 (0)  0 (0) | 4 (23.53)  0 (0)  0 (0)  2 (11.76)  2 (11.76) | 2 (6.67)  0 (0)  2 (6.67)  0 (0)  0 (0) | 3 (5.36)  0 (0)  0 (0)  0 (0)  3 (5.36) | 1 (4)  0 (0)  1 (4)  0 (0)  0 (0) | 1 (8.33)  0 (0)  0 (0)  1 (8.33)  0 (0) | 0 (0)  0 (0)  0 (0)  0 (0)  0 (0) | 3 (23.08)  0 (0)  1 (7.69)  0 (0)  2 (15.38) | 19 (30.16)  0 (0)  19 (30.16)  0 (0)  2 (3.17) | 0 (0)  0 (0)  0 (0)  0 (0)  0 (0) | 0 (0)  0 (0)  0 (0)  0 (0)  0 (0) | 2 (7.14)  0 (0)  1 (3.57)  0 (0)  1 (3.57) | 0 (0)  0 (0)  0 (0)  0 (0)  0 (0) | 3 (11.11)  0 (0)  0 (0)  0 (0)  3 (11.11) | 0 (0)  0 (0)  0 (0)  0 (0)  0 (0) |
| **Suitability for metastasis surgery**  **Performed as scheduled**  **Delayed due to COVID-19**  **Chemotherapy was continued**  **Rejected due to disease progression**  **Replaced by locoregional treatments (Yttrium Microspheres)** | 27 (5.6)  20 (4.15)  4 (0.83)  4 (0.83)  1 (0.21)  2 (0.41) | 0 (0)  0 (0)  0 (0)  0 (0)  0 (0)  0 (0) | 1 (8.33)  1 (8.33)  0 (0)  0 (0)  0 (0)  0 (0) | 3 (8.11)  0 (0)  3 (8.11)  3 (8.11)  0 (0)  0 (0) | 0 (0)  0 (0)  0 (0)  0 (0)  0 (0)  0 (0) | 4 (8.7)  4 (8.7)  0 (0)  0 (0)  0 (0)  0 (0) | 1 (5.88)  1 (5.88)  0 (0)  0 (0)  0 (0)  0 (0) | 0 (0)  0 (0)  0 (0)  0 (0)  0 (0)  0 (0) | 0 (0)  0 (0)  0 (0)  0 (0)  0 (0)  0 (0) | 0 (0)  0 (0)  0 (0)  0 (0)  0 (0)  0 (0) | 1 (8.33)  1 (8.33)  0 (0)  0 (0)  0 (0)  0 (0) | 0 (0)  0 (0)  0 (0)  0 (0)  0 (0)  0 (0) | 0 (0)  0 (0)  0 (0)  0 (0)  0 (0)  0 (0) | 10 (15.87)  9 (14.29)  1 (1.59)  1 (1.59)  0 (0)  0 (0) | 0 (0)  0 (0)  0 (0)  0 (0)  0 (0)  0 (0) | 0 (0)  0 (0)  0 (0)  0 (0)  0 (0)  0 (0) | 4 (14.29)  4 (14.29)  0 (0)  0 (0)  0 (0)  0 (0) | 0 (0)  0 (0)  0 (0)  0 (0)  0 (0)  0 (0) | 3 (11.11)  0 (0)  0 (0)  0 (0)  1 (3.7)  2 (7.41) | 0 (0)  0 (0)  0 (0)  0 (0)  0 (0)  0 (0) |

**Abbreviations**: HU, Hospital Universitario; HGU, Hospital General Universitario; CHUA, Complejo Hospitalario Universitario de A Coruña; CHUO, Complejo Hospitalario Universitario de Orense; HUCA, Hospital Universitario Central de Asturias, HUMV, Hospital Universitario Marqués de Valdecilla; HUN, Hospital Universitario de Navarra;

HUMS, Hospital Universitario Miguel Servet; VHIO, Hospital Universitario de la Vall d'Hebron y Vall d'Hebron Instituto de Oncología; ICO, Instituto Catalán de Oncología; H. San Pau, Hospital de la Santa Creu i Sant Pau; HGU de Valencia, Hospital General Universitario de Valencia; IVO, Instituto Valenciano de Oncología; HGU de Elche,

Hospital General Universitario de Elche; HGUGM, Hospital General Universitario Gregorio Marañón; HU La Paz, Hospital Universitario La Paz; HURC, Hospital Universitario Ramón y Cajal; HURS, Hospital Universitario Reina Sofía; HUVR, Hospital Universitario Virgen del Rocío.
