# Supplementary material for: Three-year survival follow-up of patients with gastrointestinal cancer treated during the COVID-19 pandemic in Spain: data from the PANDORA-TTD20 study
Source: Oncologist. 2024 Nov 16;30(8):oyae300. doi: 10.1093/oncolo/oyae300 (PMC12395236; doi:10.1093/oncolo/oyae300)
Supplement: oyae300_suppl_Supplementary_Table_S6 [file oyae300_suppl_supplementary_table_s6.docx]

**Supplementary Table 6.** Characteristics of COVID-19 episodes.

| **Characteristics of COVID-19 episodes** | **N=703 (100%)** |
| --- | --- |
| **Confirmed SARS-CoV-2 infection diagnosis** | 30 (4.2) |
| **Type of confirmed SARS-CoV-2 infection diagnosis**  **Positive PCR test**  **Positive IgG antibody test** | 27 (3.8)  3 (0.4) |
| **Diagnosis of COVID-19 pneumonia** | 11 (1.5) |
| **Hospital admission** | 10 (1.4) |
| **Admission to intensive care unit** | 0 |
| **Outcome of infection**  **Recovery**  **Sequelae**  **Death** | 25 (3.5)  3 (0.4)  2 (0.2) |
| **Status after recovering from COVID-19 infection**  **Case re-evaluated by the committee**  **Rejection of cancer treatment**  **Rejection of systemic treatment**  **Progression of cancer**  **Treatment modification** | N=28  2 (0.2)  3 (10.7)  4 (14.2)  9 (32.1)  7 (25) |
