# Supplementary material for: Three-year survival follow-up of patients with gastrointestinal cancer treated during the COVID-19 pandemic in Spain: data from the PANDORA-TTD20 study
Source: Oncologist. 2024 Nov 16;30(8):oyae300. doi: 10.1093/oncolo/oyae300 (PMC12395236; doi:10.1093/oncolo/oyae300)
Supplement: oyae300_suppl_Supplementary_Table_S7 [file oyae300_suppl_supplementary_table_s7.docx]

**Supplementary Table 7.** Management of patients with non-metastatic or metastatic cancer grouped by the type of adaptation of activity due to the COVID-19 pandemic.

| **Management of patients with non-metastatic cancer** | **Total** | **Maintained** | **Adapted** | **Suspended** |
| --- | --- | --- | --- | --- |
| **Total** | **221 (100)** | **75 (100)** | **134 (100)** | **12 (100)** |
| **Treatment and Follow-Up Management**  **Follow-Up visits**  **Treatment visits**  **Adjuvant treatment not indicated**  **Adjuvant treatment indicated**  **Prescribed despite the pandemic**  **Not prescribed due to the pandemic**  **Not prescribed for other reasons** | 121 (54.8)  100 (45.2)  21 (9.5)  79 (82.2)  74 (33.5)  1 (0.4)  4 (1.8) | 44 (58.7)  31 (41.3)  6 (8)  25 (83.3)  25 (33.3)  0 (0)  0 (0) | 68 (50.8)  66 (49.2)  15 (11.1)  51 (80.9)  46 (34.3)  1 (0.75)  4 (2.99) | 9 (75)  3 (25)  0 (0)  3 (100)  3 (25)  0 (0)  0 (0) |
| **Treatment strategies**  **Standard therapy**  **Modification of the regimen**  **Standard regimen adjusting dose**  **Standard regimen adjusting interval**  **Standard regimen adjusting dose and interval**  **Change the route of administration of any drug from IV to oral**  **Modification of visits**  **In-person and telephone visits were alternated**  **Only telephone visits were conducted**  **Visits were spaced out** | 70 (31.67)  13 (5.88)  6 (2.71)  5 (2.26)  1 (0.45)  1 (0.45)  21 (9.5)  13 (5.88)  6 (2.71)  2 (0.9) | 23 (30.67)  6 (8)  3 (4)  3 (4)  0 (0)  0 (0)  5 (6.67)  3 (4)  1 (1.33)  1 (1.33) | 45 (33.58)  6 (4.48)  2 (1.49)  2 (1.49)  1 (0.75)  1 (0.75)  15 (11.19)  9 (6.72)  5 (3.73)  1 (0.75) | 2 (16.67)  1 (8.33)  1 (8.33)  0 (0)  0 (0)  0 (0)  1 (8.33)  1 (8.33)  0 (0)  0 (0) |
| **Management of patients with metastatic cancer** | **Total** | **Maintained** | **Adapted** | **Suspended** |
| **Total** | **482 (100)** | **184 (100)** | **273 (100)** | **25 (100)** |
| **Treatment and Follow-Up Management**  **Follow-up visit**  **Treatment visit**  **Prescribed despite the pandemic**  **Not prescribed due to the pandemic**  **Not prescribed for other reasons** | 109 (22.6)  313 (64.9)  272 (56.4)  7 (1.5)  34 (7.1) | 42 (22.8)  120 (65.2)  103 (56)  1 (0.5)  16 (8.7) | 62 (22.7)  177 (64.8)  154 (56.4)  6 (2.2)  17 (6.2) | 5 (20)  16 (64)  15 (60)  0 (0)  1 (4) |
| **Reasons for not initiating or continuing systemic treatment**  **Improved supportive care as systemic treatment is contraindicated**  **Improved supportive care favored by the pandemic**  **Mixed reasons** | 10 (2.07)  8 (1.66)  23 (4.77) | 5 (2.72)  1 (0.54)  11 (5.98) | 5 (1.83)  7 (2.57)  11 (4.03) | 0 (0)  0 (0)  1 (4) |
| **Type of systemic treatment**  **First-line chemotherapy with palliative intention**  **First-line chemotherapy with intention to convert**  **Second-line chemotherapy**  **Third-line chemotherapy or beyond**  **Adjuvant chemotherapy for metastasis resection** | 126 (46.3)  40 (14.7)  67 (24.6)  31 (11.4)  8 (2.9) | 50 (27.17)  13 (7.07)  24 (13.04)  14 (7.61)  2 (1.09) | 71 (26.01)  24 (8.79)  40 (14.65)  14 (5.13)  5 (1.83) | 5 (20)  3 (12)  3 (12)  3 (12)  1 (4) |
| **Treatment strategies**  **Standard therapy**  **Modification of the regimen**  **Alternative antineoplastic agents to the standard**  **Standard regimen adjusting dose**  **Standard regimen adjusting interval**  **Standard regimen adjusting dose and interval**  **Change the route of administration of any drug from IV to oral**  **Modification of visits**  **Several cycles were scheduled without visits and without blood tests**  **In-person and telephone visits were alternated**  **Only telephone visits were conducted**  **Visits were spaced out** | 432 (89.63)  50 (10.37)  9 (1.87)  15 (3.11)  17 (3.53)  6 (1.24)  3 (0.62)  51 (10.58)  3 (0.62)  31 (6.43)  3 (0.62)  16 (3.32) | 160 (86.96)  24 (13.04)  3 (1.63)  9 (4.89)  7 (3.8)  4 (2.17)  1 (0.54)  18 (9.78)  1 (0.54)  12 (6.52)  1 (0.54)  6 (3.26) | 250 (91.6)  23 (8.4)  5 (1.83)  5 (1.83)  9 (3.3)  2 (0.73)  2 (0.73)  31 (11.36)  2 (0.73)  17 (6.23)  2 (0.73)  10 (3.66) | 22 (88)  3 (12)  1 (4)  1 (4)  1 (4)  0 (0)  0 (0)  2 (8)  0 (0)  2 (8)  0 (0)  0 (0) |
| **Suitability for metastasis surgery**  **Performed as scheduled**  **Delayed due to COVID-19**  **Chemotherapy was continued**  **Rejected due to disease progression**  **Replaced by locoregional treatments (Yttrium Microspheres)** | 27 (5.6)  20 (4.15)  4 (0.83)  4 (0.83)  1 (0.21)  2 (0.41) | 9 (4.89)  9 (4.89)  0 (0)  0 (0)  0 (0)  0 (0) | 16 (5.86)  10 (3.66)  3 (1.1)  3 (1.1)  1 (0.37)  2 (0.73) | 2 (8)  1 (4)  1 (4)  1 (4)  0 (0)  0 (0) |
