# Supplementary material for: Three-year survival follow-up of patients with gastrointestinal cancer treated during the COVID-19 pandemic in Spain: data from the PANDORA-TTD20 study
Source: Oncologist. 2024 Nov 16;30(8):oyae300. doi: 10.1093/oncolo/oyae300 (PMC12395236; doi:10.1093/oncolo/oyae300)
Supplement: oyae300_suppl_Supplementary_Table_S8 [file oyae300_suppl_supplementary_table_s8.docx]

**Supplementary Table 8.** Baseline characteristics of patients grouped by the type of adaptation due to the COVID-19 pandemic.

| **Baseline characteristics** | **Total**  **N (%)** | **Maintained** | **Adapted** | **Suspended** |
| --- | --- | --- | --- | --- |
| **Total by group** | **703 (100)** | **259 (100)** | **407 (100)** | **37 (100)** |
| **Age, mean (range)** | 66.2 (30-89.3) | 65.6 (34.6-86.4) | 66.1 (30-89.3) | 67.5 (47.5-87.3) |
| **Sex, women** | 260 (36.98) | 103 (39.77) | 142 (34.89) | 15 (40.54) |
| **Performance status, ECOG**  **0**  **1**  **2**  **3**  **4**  **Unknown** | 209 (29.73)  341 (48.51)  66 (9.39)  16 (2.28)  5 (0.71)  66 (9.39) | 68 (26.25)  136 (52.51)  23 (8.88)  7 (2.7)  2 (0.77)  23 (8.88) | 127 (31.2)  191 (46.93)  36 (8.85)  9 (2.21)  2 (0.49)  42 (10.32) | 14 (37.84)  14 (37.84)  7 (18.92)  0 (0)  1 (2.7)  1 (2.7) |
| **Comorbidities that limit systemic treatment** | 128 (18.21) | 50 (19.31) | 67 (16.46) | 11 (29.73) |
| **Patient referred from another area or health center** | 138 (19.63) | 56 (21.62) | 76 (18.67) | 6 (16.22) |
| **Primary tumor site**  **Esophagus**  **Stomach**  **Pancreas**  **Liver and bile duct**  **Colon**  **Rectum**  **Anus** | 41 (5.83)  47 (6.69)  150 (21.34)  64 (9.1)  266 (37.84)  127 (18.07)  8 (1.14) | 16 (6.18)  13 (5.02)  59 (22.78)  21 (8.11)  101 (39)  46 (17.76)  3 (1.16) | 24 (5.9)  32 (7.86)  83 (20.39)  39 (9.58)  152 (37.35)  73 (17.94)  4 (0.98) | 1 (2.7)  2 (5.41)  8 (21.62)  4 (10.81)  13 (35.14)  8 (21.62)  1 (2.7) |
| **Tumor stage**  **Non metastatic**  **Metastatic** | 221 (31.44)  482 (68.56) | 75 (28.96)  184 (71.04) | 134 (32.92)  273 (67.08) | 12 (32.43)  25 (67.57) |
| **Clinical trial participants** | 76 (10.81) | 30 (11.58) | 42 (10.32) | 4 (10.81) |
