# Supplementary material for: Three-year survival follow-up of patients with gastrointestinal cancer treated during the COVID-19 pandemic in Spain: data from the PANDORA-TTD20 study
Source: Oncologist. 2024 Nov 16;30(8):oyae300. doi: 10.1093/oncolo/oyae300 (PMC12395236; doi:10.1093/oncolo/oyae300)
Supplement: oyae300_suppl_Supplementary_Table_S9 [file oyae300_suppl_supplementary_table_s9.docx]

**Supplementary Table 9**. Overall survival according to tumor type, stage, and center.

| **Strata** | **(N/n), median OS [months] (95% CI, min. - max.)** |
| --- | --- |
| **Primary cancer site** |  |
| Esophagus, Localized | (17/13), 18.1 (95% CI, 13 - NA) |
| Esophagus, Metastatic | (24/22) 8.3 (95% CI, 6.5 - 13.6) |
| Stomach, Localized | (16/6), NA (95% CI, 22.1 - NA) |
| Stomach, Metastatic | (29/28), 9 (95% CI, 5.5 - 16.4) |
| Pancreas, Localized | (41/25), 21 (95% CI, 12 - NA) |
| Pancreas, Metastatic | (106/100), 8.9 (95% CI, 6.7 - 11.5) |
| Hepatobiliary, Localized | (12/8), 22.2 (95% CI, 12.1 - NA) |
| Hepatobiliary, Metastatic | (50/45), 9.9 (95% CI, 7.9 - 14.3) |
| Colon, Localized | (72/14), NA (95% CI, 37.3 - NA) |
| Colon, Metastatic | (187/118), 28.1 (95% CI, 19.6 - 32.8) |
| Rectum, Localized | (58/18), NA (95% CI, NA - NA) |
| Rectum, Metastatic | (67/52), 15.2 (95% CI, 9.8 - 22.4) |
| Anus, Localized | (4/3), 14.4 (95% CI, 10.9 - NA) |
| Anus, Metastatic | (3/2), 8.2 (95% CI, 3.5 - NA) |
| CHUA, Localized | None |
| CHUA, Metastatic | (24/20), 8.2 (95% CI, 7.3 - 15.7) |
| CHUO, Localized | (3/2), 6.9 (95% CI, 6.7 - NA) |
| CHUO, Metastatic | (12/11), 19.6 (95% CI, 13.5 - NA) |
| HUCA, Localized | (10/2), NA (95% CI, NA - NA) |
| HUCA, Metastatic | (37/28), 14.6 (95% CI, 9.6 - 33.8) |
| HUMV, Localized | (4/4), 25.1 (95% CI, 0.4 - NA) |
| HUMV, Metastatic | (10/10), 13.3 (95% CI, 8.2 - NA) |
| HU Donostia, Localized | (23/5), NA (95% CI, NA - NA) |
| HU Donostia, Metastatic | (46/39), 13.2 (95% CI, 6.8 - 20.7) |
| HUN, Localized | (15/2), NA (95% CI, NA - NA) |
| HUN, Metastatic | (16/10), 21.8 (95% CI, 12.4 - NA) |
| HUMS, Localized | (7/3), NA (95% CI, 10.9 - NA) |
| HUMS, Metastatic | (29/23), 14.1 (95% CI, 9.4 - 18.4) |
| VHIO, Localized | (15/10), 20.5 (95% CI, 13.9 - NA) |
| VHIO, Metastatic | (56/46), 9.5 (95% CI, 8.1 - 17.2) |
| ICO, Localized | (32/9), NA (95% CI, NA - NA) |
| ICO, Metastatic | (25/16), 9.9 (95% CI, 5.8 - NA) |
| H. San Pau, Localized | (15/5), NA (95% CI, 31.9 - NA) |
| H. San Pau, Metastatic | (12/10), 10.6 (95% CI, 6.9 - NA) |
| HGU de Valencia, Localized | (14/1), NA (95% CI, NA - NA) |
| HGU de Valencia, Metastatic | (7/4), 31.3 (95% CI, 3.7 - NA) |
| IVO, Localized | (5/2), NA (95% CI, 37.3 - NA) |
| IVO, Metastatic | (25/21), 17.7 (95% CI, 9.7 - 31.9) |
| HGU de Elche, Localized | (6/0), NA (95% CI, NA - NA) |
| HGU de Elche, Metastatic | (13/11), 5.5 (95% CI, 2 - NA) |
| HGUGM, Localized | (15/8), 16.4 (95% CI, 12.1 - NA) |
| HGUGM, Metastatic | (62/46), 16.8 (95% CI, 13.1 - 28.1) |
| HU. La Paz, Localized | (8/4), 25.1 (95% CI, 15 - NA) |
| HU. La Paz, Metastatic | (26/22), 10.4 (95% CI, 7.3 - 27.3) |
| HURS, Localized | (23/14), 28.5 (95% CI, 18.6 - NA) |
| HURS, Metastatic | (28/19), 13.8 (95% CI, 10.9 - NA) |
| HUVR, Localized | (15/15), 6.2 (95% CI, 4.8 - 15.8) |
| HUVR, Metastatic | (22/22), 9.2 (95% CI, 6.4 - 13.4) |
| Hospital Regional Universitario de Málaga, Localized | (10/1), NA (95% CI, NA - NA) |
| Hospital Regional Universitario de Málaga, Metastatic | (16/9), 31.3 (95% CI, 17.9 - NA) |

**Abbreviations**: CI, Confidence Interval; max., maximum; min., mínimum; NA, Not Achieved; CHUA, Complejo Hospitalario Universitario de A Coruña; CHUO, Complejo Hospitalario Universitario de Orense; HUCA, Hospital Universitario Central de Asturias, HUMV, Hospital Universitario Marqués de Valdecilla; HUN, Hospital Universitario de Navarra; HUMS, Hospital Universitario Miguel Servet; VHIO, Hospital Universitario de la Vall d'Hebron y Vall d'Hebron Instituto de Oncología; ICO, Instituto Catalán de Oncología; H. San Pau, Hospital de la Santa Creu i Sant Pau; HGU de Valencia, Hospital General Universitario de Valencia; IVO, Instituto Valenciano de Oncología; HGU de Elche, Hospital General Universitario de Elche; HGUGM, Hospital General Universitario Gregorio Marañón; HU La Paz, Hospital Universitario La Paz; HURC, Hospital Universitario Ramón y Cajal; HURS, Hospital Universitario Reina Sofía; HUVR, Hospital Universitario Virgen del Rocío.

Note: Survival data for the HURC have not been included as follow-up data are not available.

.
