# Supplementary material for: Three-year survival follow-up of patients with gastrointestinal cancer treated during the COVID-19 pandemic in Spain: data from the PANDORA-TTD20 study
Source: Oncologist. 2024 Nov 16;30(8):oyae300. doi: 10.1093/oncolo/oyae300 (PMC12395236; doi:10.1093/oncolo/oyae300)
Supplement: oyae300_suppl_Supplementary_Figure_S1 [file oyae300_suppl_supplementary_figure_s1.pdf]

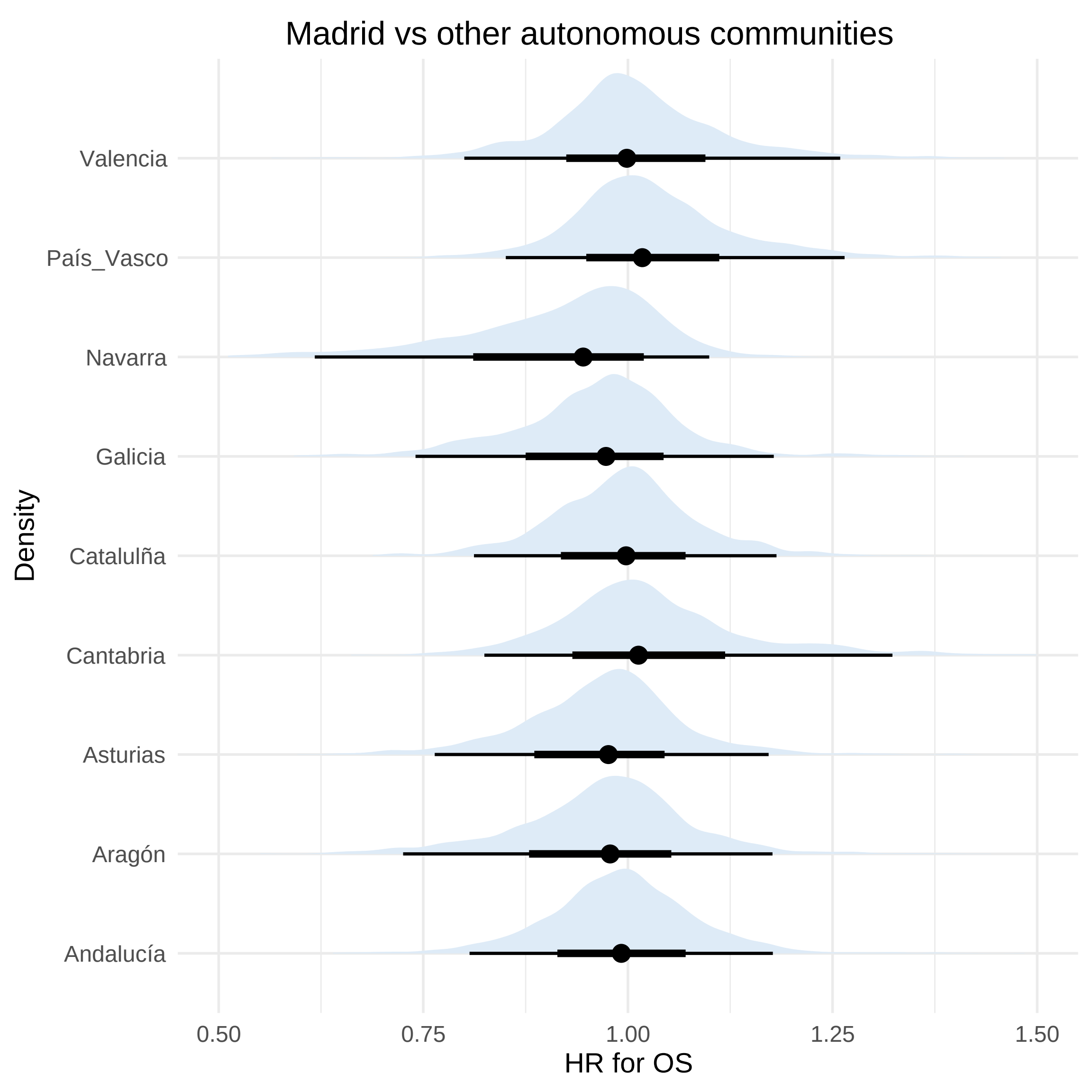
Madrid vs other autonomous communities
Valencia
País_Vasco
Navarra
Galicia
Catalulña
Cantabria
Asturias
Aragón
Andalucía
Density
0.50
0.75
1.00
1.25
1.50
HR for OS
