# Supplementary material for: Three-year survival follow-up of patients with gastrointestinal cancer treated during the COVID-19 pandemic in Spain: data from the PANDORA-TTD20 study
Source: Oncologist. 2024 Nov 16;30(8):oyae300. doi: 10.1093/oncolo/oyae300 (PMC12395236; doi:10.1093/oncolo/oyae300)
Supplement: oyae300_suppl_Supplementary_Figure_S2 [file oyae300_suppl_supplementary_figure_s2.pdf]

Covid−19 cumulative incidence

Measure: cumulative incidence

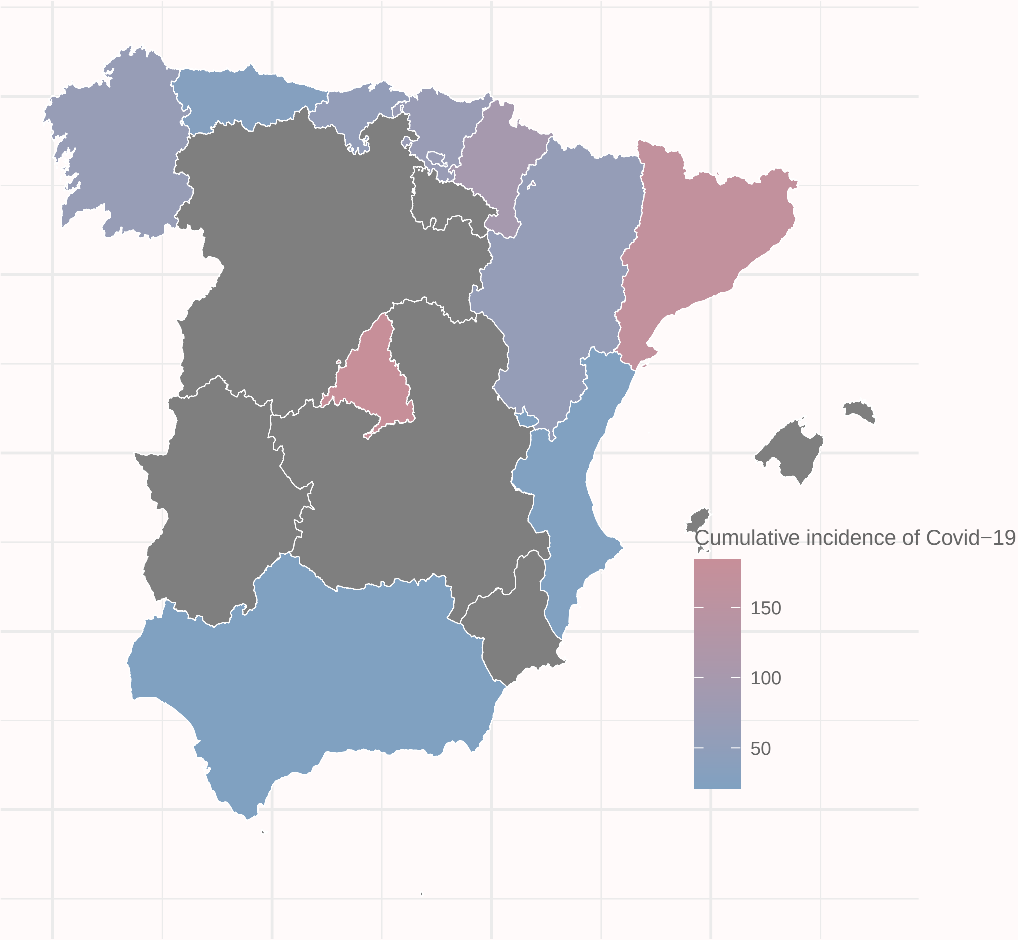

Source: Ministry of Health of Spain. Data updated on April 27, 2020.
