# Supplementary material for: Three-year survival follow-up of patients with gastrointestinal cancer treated during the COVID-19 pandemic in Spain: data from the PANDORA-TTD20 study
Source: Oncologist. 2024 Nov 16;30(8):oyae300. doi: 10.1093/oncolo/oyae300 (PMC12395236; doi:10.1093/oncolo/oyae300)
Supplement: oyae300_suppl_Supplementary_Table_S2 [file oyae300_suppl_supplementary_table_s2.docx]

**Supplementary Table 2**. R code for data analysis.

# load the libraries

library(readxl)

library(lubridate)

library(janitor)

library(dplyr)

library(survival)

library(raster)

library(rnaturalearth)

library(ggplot2)

library(ggspatial)

library(sf)

library(sp)

library(spatsurv)

library(stringr)

library(survminer)

library(ggplot2)

library(purrr)

library(viridis)

# load the dataset

v <- read_excel("C:/Users/Alberto/Desktop/pandora ttd/visita0 PACIENTES con datos seguimiento.xlsx")

# KM estimator

modelo_localiza_estadio <- survfit(Surv(tiempo_muerte, die_total) ~ localiza + estadio, data = v)

modelo_codcentro <- survfit(Surv(tiempo_muerte, die_total) ~ centro, data = v)

modelo_codcentro_estadio <- survfit(Surv(tiempo_muerte, die_total) ~ centro + estadio, data = v)

modelo_localiza_colon_recto <- survfit(Surv(tiempo_muerte, die_total) ~ localiza, data = v[v$localiza %in% c("colon", "recto"),])

modelo_estadio_metastasico <- survfit(Surv(tiempo_muerte, die_total) ~ estadio, data = v[v$estadio == "metastasico",])

resumen <- lapply(

list(modelo_localiza_estadio, modelo_codcentro_estadio),

function(x) {

data.frame(

strata=rownames(summary(x)$table),

N = x$n,

n = as.numeric(summary(x)$table[,4]),

Mediana = surv_median(x)[2],

Intervalo_Confianza_Inferior = surv_median(x)[3],

Intervalo_Confianza_Superior = surv_median(x)[4]

)

}

)

nombres_modelos <- c( "localiza_estadio", "codcentro_estadio")

df <- map2_df(resumen, nombres_modelos, ~ cbind(.x, modelo = .y))

df$modelo <- factor(df$modelo, levels = nombres_modelos)

parts <- strsplit(df$strata, split = ", ")

df$strata <- sapply(parts, function(x) {

str_list = sapply(strsplit(x, split = "="), function(y) y[2])

paste(unlist(str_list), collapse = ", ")

})

if(length(df$strata) != nrow(df)){

df$strata <- rep(df$strata, length.out = nrow(df))

}

max_limit <- max(na.omit(c(df$upper, df$median)))

df$color <- rep(c("color1", "color2"), len = nrow(df))

df$color <- factor(df$color)

# Creamos una columna con el texto que queremos mostrar

df$texto <- paste("(", df$N, "/", df$n, ") ", round(df$median, 1), " months (95% CI, ", round(df$lower, 1), "-", round(df$upper, 1), ")")

# Gráfico

ggplot(df, aes(y = strata, x = median, color = color)) +

geom_segment(aes(x = lower, xend = median, yend = strata)) +

geom_point(size = 3) +

geom_segment(data = subset(df, !is.na(upper)), aes(xend = upper, yend = strata)) +

geom_segment(data = subset(df, is.na(upper)), aes(xend = max_limit, yend = strata), linetype = "dashed") +

facet_wrap(~ modelo, scales = "free", labeller = as_labeller(c(`localiza_estadio` = "Tumor site by stage", `codcentro_estadio` = "Center by stage")))+

theme(axis.text.y = element_text(angle = 0, vjust = 0.5, hjust = 1)) +

theme_minimal() +

geom_segment(data = subset(df, is.na(median)), aes(x = min(df$lower, na.rm=TRUE), xend = max_limit, yend = strata), linetype = "dotted")+

labs(y = "Strata", x = "Median survival since April 2020 (months)", color = "Color") + theme(legend.position="none") +

scale_x_continuous(breaks = round(seq(min(df$lower, na.rm=TRUE), max(df$upper, na.rm = TRUE), by = 3),0)) +scale_color_aaas() # land 6x12

# map of spain ---> https://rquer.netlify.app/static_map/mapas_spain/

library(broom)

library(rgdal)

library(sf)

shapefile_provincias <-

readOGR("C:/Users/Alberto/Desktop/pandora ttd/ComunidadesAutonomas_ETRS89_30N/Comunidades_Autonomas_ETRS89_30N.shp")

data_provincias <- tidy(shapefile_provincias)

nombres_provincias <- data.frame(shapefile_provincias$Texto)

nombres_provincias$id <- as.character(seq(0, nrow(nombres_provincias)-1))

data_provincias_mapa <- left_join(data_provincias, nombres_provincias, by = "id")

data_provincias_mapa %>%

ggplot() +

geom_polygon(aes( x= long, y = lat, group = group),

fill = "violetred4",

color = "white") +

theme_minimal() +

theme(

axis.line = element_blank(),

axis.text = element_blank(),

axis.title = element_blank(),

axis.ticks = element_blank(),

panel.background = element_rect(colour= "darkgrey", size= 0.5)) +

ggtitle("Provincias Españolas")

################################################# the model

library(spBayesSurv)

library(R2BayesX)

s<-data.frame(time=m$tiempo_muerte,

event=m$die_total,

id_c=m$codcentro,

centro=m$centro,

x=m$xcoord,

y=m$ycoord,

ecog=factor(m$ecog),

localiza=m$localiza,

linea=m$linea,

estadio=factor(m$estadio),

id_f= as.numeric(m$codcentro),

adaptacion= factor (m$a12_v1))

s<-subset(s, time>0)

coords <- s %>%

distinct(centro, .keep_all = TRUE)

co<-c(coords[,1], coords[,2])

mat1 <- matrix(co,ncol=2,byrow=FALSE)

mat_num <- matrix(as.numeric(mat1), # Convert to numeric matrix

ncol = ncol(mat1))

mat_num

coords <- as.matrix(coords[, c("x", "y", "centro","id_f")])

coords[,1] <- as.numeric(str_trim( coords[,1]))

coords2<-as.numeric(coords[,2])

coords1<-as.numeric(coords[,1])

coords<-cbind(coords1,coords2,coords)

coords3<- data.frame(coords)

coords_sf <- st_as_sf(coords3, coords = c("coords1", "coords2"), crs = 4326)

# Transformar a UTM con la zona 30

coords_utm <- st_transform(coords_sf, crs = "+proj=utm +zone=30 +ellps=WGS84 +datum=WGS84 +units=m +no_defs")

nuevo_df <- st_coordinates(coords_utm) %>%

as.data.frame() %>%

setNames(c("x", "y"))

coords5 <- s %>%

distinct(centro, .keep_all = TRUE) %>%as.vector()

coords2<- data.frame(x= nuevo_df[,1], y = nuevo_df[,2], centro=coords5$centro, codcentro=coords5$id_f) # esto se aplica tras modelo

set.seed(1)

mcmc=mcmc=list(nburn=3000, nsave=2000, nskip=0, ndisplay=500)

prior <- list(maxL = 15,

nknots = 10)

ptm<-proc.time()

s=s[order(s$id_f),]

s$ID <- 1:nrow(s)

co<-c(coords2[,1], coords2[,2])

mat1 <- matrix(co,ncol=2,byrow=FALSE)

mat_num <- matrix(as.numeric(mat1), # Convert to numeric matrix

ncol = ncol(mat1))

mat_num

res1=survregbayes(formula=Surv(time,event)~linea+localiza+estadio+ecog+frailtyprior("grf",id_f),

data=s,survmodel="PH", dist="weibull",Coordinates = mat_num,

mcmc=mcmc,prior=prior)

saveRDS(res1, file = "C:/Users/Alberto/Desktop/pandora ttd/centros_covid", ascii = FALSE, version = NULL,

compress = TRUE, refhook = NULL)

frail <- round(apply(res1$v, 1, mean), 3)

nclust <- 5

frail.cluster <- cut(frail, breaks = nclust)

frail.names <- names(table(frail.cluster))

rbPal <- colorRampPalette(c('blue', 'red'))

frail.colors <- rbPal(nclust)[as.numeric(frail.cluster)]

coords2<- data.frame(x= mat_num[,1], y = mat_num[,2], frail=exp(frail), centro=coords2$centro) # esto se aplica tras modelo para pintar el mapa

coords2$frail = round(coords2$frail,2)

sfit1=summary(res1)

sfit1

library(sf)

library(ggrepel)

mapa_centros <- data_provincias_mapa %>% filter(shapefile_provincias.Texto != "Canarias") %>%

ggplot() +

geom_polygon(aes( x= long, y = lat, group = group),

fill = "lightgray",

color = "white") +

geom_point(data = coords2, aes(x = x, y = y, size = frail, color = frail, alpha=0.5)) +

scale_color_viridis_c(option = "plasma")+

theme_minimal() +

geom_text_repel(data = coords2,

aes(x = x, y = y, label = centro),

nudge_x = 70000, nudge_y = 70000,

segment.size = 1,

segment.color = "black", max.iter=7000) +

scale_size_continuous(range = c(5, 35)) + # Ajusta el rango de tamaño de las burbujas según tus preferencias

theme(

axis.line = element_blank(),

axis.text = element_blank(),

axis.title = element_blank(),

axis.ticks = element_blank(),

panel.background = element_rect(colour= "darkgrey", size= 0.5)) +

ggtitle("Provincias Españolas")+ labs(

title = "Mortality risk",

subtitle = "Measure: relative hazards",

caption = "Source: TTD",

fill = "Relative hazard"

)

mapa_centros

saveRDS(mapa_centros, file = "C:/Users/Alberto/Desktop/pandora ttd/mapa_centros", ascii = FALSE, version = NULL,

compress = TRUE, refhook = NULL)

#################################################

#################################################

#################################################

#################################################

# el modelo regional

#################################################

#################################################

#################################################

#################################################

library(spBayesSurv)

library(R2BayesX)

v$seguimiento <- v$d101_v0

v$seguimiento[is.na(v$seguimiento)]<-0

m<-subset(v, seguimiento=="0")

m$d18_v0<-ifelse(m$d18_v0=='4'|m$d18_v0=='5',"3",m$d18_v0)

m$linea <- factor(m$d18_v0)

m$ecog<- as.numeric(m$c3_v0)-1

m$ecog<-ifelse(m$ecog==4,3,m$ecog)

m$ecog<-factor(m$ecog)

s<-data.frame(time=m$tiempo_muerte,

event=m$die_total,

x=m$xcoord,

y=m$ycoord,

ecog=factor(m$ecog),

localiza=m$localiza,

linea=m$linea,

estadio=factor(m$estadio),

id= m$id)

s$x<-as.numeric(str_trim(s$x, side = "right"))

s$y<-as.numeric(str_trim(s$y, side = "right"))

s=s[order(s$id),]

s<-subset(s, time>0)

set.seed(1)

mcmc=mcmc=list(nburn=3000, nsave=2000, nskip=0, ndisplay=500)

prior=list(maxL=19)

ptm<-proc.time()

# el modelo aft

res1b=survregbayes(formula=Surv(time,event)~linea+localiza+estadio+ecog+frailtyprior("iid",id),data=s,survmodel="AFT", dist="weibull",

mcmc=mcmc,prior=prior)

saveRDS(res1b, file = "C:/Users/Alberto/Desktop/pandora ttd/regiones_covid", ascii = FALSE, version = NULL,

compress = TRUE, refhook = NULL)

sfit1=summary(res1b)

sfit1

guardar_modelo<- as.data.frame(sfit1$coeff)

guardar_modelo$HR <-exp(guardar_modelo[,1])

guardar_modelo$se <-guardar_modelo[,3]

guardar_modelo$HR <-exp(guardar_modelo[,4])

guardar_modelo$HR <-exp(guardar_modelo[,5])

guardar_modelo <-cbind(rownames(sfit1$coeff), guardar_modelo)

library(openxlsx)

write.xlsx(guardar_modelo,

file = "C:/Users/Alberto/Desktop/pandora ttd/modelo2.xlsx",

colNames = TRUE, borders = "surrounding")

frail0=(rowMeans(res1$v))

frail=frail0

values=cbind(sort(unique(v$id)),frail)

op<-par(no.readonly=TRUE)

#el modelo de proportional hazards

res1c=survregbayes(formula=Surv(time,event)~linea+localiza+estadio+ecog+frailtyprior("iid",id),data=s,survmodel="PH", dist="weibull",

mcmc=mcmc,prior=prior)

saveRDS(res1c, file = "C:/Users/Alberto/Desktop/pandora ttd/regiones_covid2", ascii = FALSE, version = NULL,

compress = TRUE, refhook = NULL)

res1c<-readRDS(file = "C:/Users/Alberto/Desktop/pandora ttd/regiones_covid2")

sfit1=summary(res1c)

sfit1

frail0=(rowMeans(res1c$v))

frail=frail0

values=cbind(sort(unique(v$id)),frail) # id2= asturias 3p, id5= cataluña 5posicion

op<-par(no.readonly=TRUE)

mapa <- as.tibble(values)

names(mapa)<-c("id","frail")

mapa$id <- as.character(mapa$id)

mapa_grafico <- data_provincias_mapa %>%

left_join(mapa, by= "id")

mapa_grafico %>%

filter(shapefile_provincias.Texto != "Canarias") %>%

ggplot(aes(x = long, y = lat, group = group)) +

geom_polygon(aes(fill = exp(frail)), color = "white", size = 0.2) +

scale_fill_gradient(low = hcl(240, 35, 65), high = hcl(0, 35, 65))+

labs(

title = "Mortality risk",

subtitle = "Measure: relative hazards",

caption = "Source: TTD",

fill = "Relative hazard"

) +

theme_minimal() +

theme(

axis.line = element_blank(),

axis.text = element_blank(),

axis.title = element_blank(),

axis.ticks = element_blank(),

plot.background = element_rect(fill = "snow", color = NA),

panel.background = element_rect(fill = "snow", color = NA),

plot.title = element_text(size = 16, hjust = 0),

plot.subtitle = element_text(size = 12, hjust = 0),

plot.caption = element_text(size = 8, hjust = 1),

legend.title = element_text(color = "grey40", size = 8),

legend.text = element_text(color = "grey40", size = 7, hjust = 0),

legend.position = c(0.93, 0.3),

plot.margin = unit(c(0.5, 2, 0.5, 1), "cm")

)

# voy a tratar de sumar este mapa con el previo de los bubbles

library(scales)

color_transparente = alpha("gainsboro", 1)

mapa_grafico %>%

filter(shapefile_provincias.Texto != "Canarias") %>%

ggplot(aes(x = long, y = lat)) +

geom_polygon(aes(group = group, fill = exp(frail)), color = "white", size = 0.2, alpha = 0.5) + # Añadimos alpha = 0.5 aquí

scale_fill_continuous(na.value = "#DCDCDCFF") +

scale_color_viridis_c(option = "magma") +

geom_point(data = coords2, aes(x = x, y = y, size = frail, color = frail, alpha=0.5)) +

scale_color_gradient2(low = viridis_pal(option = "inferno")(3)[1],

mid = viridis_pal(option = "inferno")(3)[2],

high = "orange",

midpoint = median(coords2$frail, na.rm = TRUE)) + theme_minimal() +

geom_text_repel(data = coords2,

aes(x = x, y = y, label = centro),

nudge_x = 70000, nudge_y = 70000,

segment.size = 1,

segment.color = "black", max.iter=7000) +

scale_size_continuous(range = c(5, 35)) +

theme(

axis.line = element_blank(),

axis.text = element_blank(),

axis.title = element_blank(),

axis.ticks = element_blank(),

panel.background = element_rect(colour= "gainsboro", size= 0.5)) +

ggtitle("Provincias Españolas") + labs(

title = "Mortality risk by Autonomous Comunity & Center",

subtitle = "Measure: relative hazards",

caption = "Source: TTD",

fill = "Relative hazard"

) # 12 x 13 landscale
